# Fc gamma receptors are expressed in the developing rat brain and activate downstream signaling molecules upon cross-linking with immune complex

**DOI:** 10.1186/s12974-017-1050-z

**Published:** 2018-01-06

**Authors:** Marianna Stamou, Ana Cristina Grodzki, Marc van Oostrum, Bernd Wollscheid, Pamela J. Lein

**Affiliations:** 1 Department of Molecular Biosciences, School of Veterinary Medicine, University of California, 1089 Veterinary Medicine Drive, Davis, CA 95616 USA; 2 Department of Health Sciences and Technology, Institute of Molecular Systems Biology, ETH Zurich, 8093 Zürich, Switzerland

**Keywords:** Fc gamma receptor (FcγR), Neurons, Astrocytes, Hippocampus, Cortex, IgG immune complex (IgG-IC), Cross-linking, Neurodevelopment

## Abstract

**Background:**

Exposure of the developing brain to immune mediators, including antibodies, is postulated to increase risk for neurodevelopmental disorders and neurodegenerative disease. It has been suggested that immunoglobulin G-immune complexes (IgG-IC) activate Fc gamma receptors (FcγR) expressed on neurons to modify signaling events in these cells. However, testing this hypothesis is hindered by a paucity of data regarding neuronal FcγR expression and function.

**Methods:**

FcγR transcript expression in the hippocampus, cortex, and cerebellum of neonatal male and female rats was investigated ex vivo and in mixed cultures of primary hippocampal and cortical neurons and astrocytes using quantitative PCR analyses. Expression at the protein level in mixed cultures of primary hippocampal and cortical neurons and astrocytes was determined by immunocytochemistry, western blotting, proteotype analysis, and flow cytometry. The functionality of these receptors was assessed by measuring changes in intracellular calcium levels, Erk phosphorylation, and IgG internalization following stimulation with IgG-immune complexes.

**Results:**

*FcgrIa*, *FcgrIIa*, *FcgrIIb*, *FcgrIIIa*, and *Fcgrt* transcripts were detectable in the cortex, hippocampus, and cerebellum at postnatal days 1 and 7. These transcripts were also present in primary hippocampal and cortical cell cultures, where their expression was modulated by IFNγ. Expression of FcγRIa, FcγRIIb, and FcγRIIIa, but not FcγRIIa or FcRn proteins, was confirmed in cultured hippocampal and cortical neurons and astrocytes at the single cell level. A subpopulation of these cells co-expressed the activating FcγRIa and the inhibitory FcγRIIb. Functional analyses demonstrated that exposure of hippocampal and cortical cell cultures to IgG-IC increases intracellular calcium and Erk phosphorylation and triggers FcγR-mediated internalization of IgG.

**Conclusions:**

Our data demonstrate that developing neurons and astrocytes in the hippocampus and the cortex express signaling competent FcγR. These findings suggest that IgG antibodies may influence normal neurodevelopment or function via direct interactions with FcγR on non-immune cells in the brain.

**Electronic supplementary material:**

The online version of this article (10.1186/s12974-017-1050-z) contains supplementary material, which is available to authorized users.

## Background

Emerging evidence that neurons express classic immune molecules such as complement receptors and MHC class I proteins has spurred significant research interest in the role of immune molecules in CNS development and disease [1–6]. A class of immune molecules that is receiving increased attention in this context is the Fc gamma receptors (FcγR) [7–9]. Neuronal FcγR have been shown to contribute to kainic-acid- and amyloid-β-mediated neurotoxicity in the brain [10, 11], but have also been suggested to play a role in normal neurodevelopment [12, 13]. More recently, neuronal uptake of antibodies against intracellular neuronal proteins has been shown to occur in the adult brain and to be mediated primarily via clathrin-dependent FcγR endocytosis, with limited microglial involvement, ultimately leading to target protein degradation [14, 15]. IgG antibodies against viral or self-antigens are often found in the developing human brain and at least a subset of these has been linked to increased risk for neurodevelopmental disorders [16–26]. However, the mechanisms by which immunoglobulins interfere with neurodevelopment remain speculative. Considering that IgG is the cognate ligand for FcγR, expression of FcγR on neurons and macroglia in the developing brain might provide a plausible biological mechanism linking IgG antibodies found in the developing brain to adverse neurodevelopmental outcomes [27–29].

FcγR are transmembrane glycoproteins expressed on effector cells of the innate immune system that recognize the constant Fc (fragment crystallizable) domain of immunoglobulin G (IgG) [30, 31]. Cross-linking of FcγR by IgG immune complexes (IgG-IC) triggers IgG internalization, cytokine release, phagocytosis, antibody-dependent cytotoxicity, regulation of antibody production, and gene transcription [32]. The major FcγR family members are FcγRIa (CD64), FcγRIIb (CD32), and FcγRIIIa (CD16). “Activating” receptors FcγRIa and FcγRIIIa signal via an immunoreceptor tyrosine-based activation motif (ITAM). Their cross-linking by IgG-IC increases intracellular calcium, activates cellular kinases, and triggers transcription of genes involved in host immune responses. These signals are attenuated by the “inhibiting” receptor (FcγRIIb), which carries an immunoreceptor tyrosine-based inhibition motif (ITIM) sequence. Each FcγR has different affinity for individual IgG isotypes, and the co-expression of activating and inhibitory FcγR results in a complex threshold for cellular activation that is dependent on the relative expression of FcγR subtypes, their posttranslational modifications, and the IgG isotype composition of their IgG-IC ligand [33–35]. An exception is the neonatal Fc receptor (FcRn). FcRn is an MHC class I molecule, which is thought to protect IgG from catabolism through bidirectional transcytosis of IgG across endothelial and epithelial cells [36].

Microglia, which are the innate immune cells of the brain, are known to express FcγR, which mediate microglial phagocytosis of dying and stressed cells [37, 38]. However, emerging evidence suggests that cells in the brain that are not involved in immune surveillance, specifically, neurons and macroglia, may also express FcγR. *FcgRI*, *II*, *III*, and *IV* transcripts have been reported in mouse cortical and hippocampal neurons and astrocytes [39]. At the protein level, detection of FcγRI and FcγRII but not FcγRIII were reported in a recent whole proteome analysis of the adult mouse brain, while FcγRI but neither FcγRII nor FcγRIII were detected in samples from DIV10 and DIV15 mouse cortical cells [40]. There are also reports of FcγR expression in mouse cortical astrocytes [40, 41] and FcγRIIb expression on mouse parvalbumin neurons, Purkinje cells, and Bergman glia [10, 13]. In humans, FcγRIIb expression has been reported in the hippocampus [42]. However, to date, very few studies have comprehensively profiled FcγR in the developing brain or investigated the signaling competence of neuronal and macroglial FcγR [43, 44].

The objectives of this study, therefore, were to characterize expression and cell surface presence of FcγR in neurons and astrocytes of the developing rat brain and to assess whether immune complexes added to primary cultures of neurons and astrocytes trigger activation of canonical FcγR downstream signaling.

## Methods

### Experimental design

The in vivo and in vitro experiments were conducted at the developmental times illustrated in the schematic below:
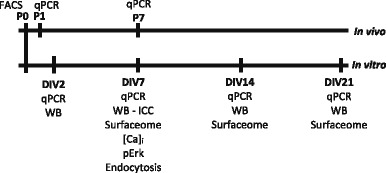


### Animals

For the majority of studies, which were performed at UC Davis (Davis, CA), timed pregnant female Sprague Dawley rats were purchased from Charles River Laboratories and housed individually in standard plastic cages with Alpha-Dri bedding (Shepherd Specialty Papers) in a temperature-controlled room (22 ± 2 °C) on a normal 12 h light/dark cycle. Food and water were provided ad libitum.

For the surfaceome analyses, which were performed at the ETH (Zurich, Switzerland), Sprague Dawley rats were purchased from Charles River Laboratories and group housed (three per cage) in Allentown individually ventilated cages with Lignocel bedding (JRS - Rettenmaier) in a temperature-controlled room (21 ± 2 °C) on a normal 12-h light/dark cycle. Food and water (Kliba) were provided ad libitum.

### Primary neuronal cell culture

Primary cultures of dissociated hippocampal or cortical cells were prepared and maintained as previously described [45]. Briefly, hippocampi and neocortices from pooled postnatal day 0 (P0) or P1 male and female rat pups were dissociated and plated onto tissue culture plastic or on glass coverslips precoated with poly-L-lysine (Sigma), at different densities depending on the endpoint to be analyzed (detailed information provided in the respective method section). Cells were maintained in Neurobasal A media supplemented with B27 and 2 mM Glutamax (all from Life Technologies), except for cultures used in the proteomics experiments, for which cells were maintained in Neural Q supplemented with GS21 (both from Sigma). Cytosine-D-arabinofuranoside (araC; 5 μM, Sigma) was added to the medium on day in vitro (DIV) 4 to prevent overgrowth of astrocytes. Half the medium was replaced with fresh media (without araC) twice weekly.

### Rat peritoneal cells

Resident peritoneal macrophages were collected from adult female Sprague Dawley rats, plated at a density of 2 × 10^5^ cells/cm^2^ and maintained as described [46]. Cells were plated on tissue culture plastic (Thermo Fisher Scientific) and allowed to adhere for 1 h at 37 °C in a 5% CO_2_ humidified incubator. Non-adherent cells were removed by washing the plates twice with warm RPMI 1640 medium (Gibco). This procedure enriches remaining adherent cells for macrophages (~ 95%) [47]. Using this macrophage-enriched population of peritoneal cells, specificity of the anti-FcγRI (anti-CD64) and anti-FcγRII (anti-CD32) antibodies was determined by confirming lack of CD64/CD32 immunolabeling on CD3+ cells and positive CD64/CD32 immunolabeling on CD3- cells.

### Quantitative real-time PCR (qPCR)

To assess *Fcgr* transcript levels in vivo, rat pups were euthanized on P1 or P7 (*n* = 3 pups of each sex and age from the first litter; *n* = 3 female and 3 male pups at P1 and 3 female and 2 male pups at P7 from the second litter). Sex was determined by anogenital distance. P1 pups were euthanized by decapitation; P7 pups were placed on a cloth-lined Petri dish on top of ice to induce hypothermic anesthesia, and then decapitated, and liver, spleen, and brain were removed for further analysis. To assess in vitro *F*c*gr* expression, cultured hippocampal or cortical cells were harvested from P1 male and female pups (*n* = 6 cultures per experimental group with 3 cultures each from 2 independent dissections; cells from both sexes were pooled). Immediately following euthanasia, the brain (microdissected into hippocampus, cortex, and cerebellum, after carefully removing meninges), liver, and spleen were harvested and placed in RNAlater overnight (Life Technologies). The following day, RNAlater was removed and the samples stored at − 80 °C until further analysis. To assess in vitro expression of *F*c*gr*, cell lysates were collected from hippocampal and cortical neuron-glia co-cultures (seeded at a density of 0.5–1 × 10^5^ cells/cm^2^) on DIV 2, 7, 14, and 21. To investigate the effects of IFNγ on *Fcgr* transcript expression, cortical cell cultures were exposed to recombinant rat IFNγ (100 ng/ml, Peprotech) added to the culture medium on DIV 1 for 24 or 48 h (3 cultures derived from each of 6 independent dissections were used). Cells were lysed in Buffer RLT (RNeasy Mini Kit, Qiagen), lysates homogenized using QIAShredder spin columns (Qiagen), and homogenates stored at − 80 °C until further processed.

Extraction of total RNA and synthesis of cDNA from brain tissues or cell cultures were performed as previously described [48]. cDNA was loaded in duplicate in MicroAmp Optical 384-well plates (Life Technologies), and qPCR was performed at the Real-Time PCR Research and Diagnostics Core Facility at UC Davis using Taqman technology on a 7900HT Fast Real-Time PCR System (Applied Biosystems). Primer and probe sets, designed to span an exon-exon junction, were obtained from Integrated DNA Technologies (IDT). Gene accession numbers and primer/probe set product number and amplification efficiencies are presented in Table 1.

**Table 1:** Rat *Fcgr* primer and probe sets

Assay ID^a^	Gene	Gene accession number	Amplification efficiency
Rn.PT.53a.13499403	*FcgrIa*	NM_001100836	0.97
Rn.PT.53a.37633364	*FcgrIIa*	NM_053843	0.90
Rn.PT.53a.36530803	*FcgrIIb*	NM_175756	0.80
Rn.PT.53a.8706390	*FcgrIIIa*	NM_207603	0.79
Rn.PT.53a.35905066	*Fcgrt*	NM_033351	0.86
Rn.PT.42.8715862	*Pgk1*	NM_053291	0.86

*Pgk1* was used as a reference gene

^a^All primers and probes were obtained from Integrated DNA Technologies (IDT)

Specific amplification of products of the desired length was verified using gel electrophoresis (not shown). qPCR results were analyzed using the Applied Biosystems 7900HT Fast Real-Time PCR System software (SDS, Thermo Fisher Scientific; version 2.4) to obtain Ct values [49]. Across all qPCR experiments, each sample was quantified in duplicate, and duplicate Ct values were averaged for analysis of results.

Relative differences in gene expression between experimental groups were calculated as described previously [48]. Transcript levels were normalized to the concentration of the reference gene *Pgk1* [48, 50], and relative expression ratios were calculated by the Pfaffl method [51] using REST 2009 software (Qiagen). Consistent with prior studies demonstrating that Pgk1 is a stably expressed gene in the brain [50, 52], expression levels of Pgk1 were not different between experimental conditions.

### Western blotting

Cortical and hippocampal cell cultures (seeded at a cell density of 1 × 10^5^ cells/cm^2^) were lysed in ice-cold lysis buffer [50 mM Tris, pH 8.0, 150 mM NaCl, 1% Triton X-100, 0.1% sodium dodecyl sulfate (SDS), 0.5% sodium deoxycholate, and 1X Halt Protease and Phosphatase Inhibitor Cocktail (Thermo Fisher Scientific)]. The protein concentration of cell lysates was determined using the Pierce BCA Protein Assay Kit (Thermo Fisher Scientific), and 30 μg of protein from each sample was separated by SDS PAGE using 10% Bolt Bis-Tris Plus Gels (Invitrogen), transferred onto PVDF membranes using the iBlot 2 dry transfer system, and immunoblotted using the iBind Western System (Life Technologies). Proteins were identified based on expected molecular weight of immunoreactive bands as indicated in the product information provided by the supplier from which antibodies were purchased: 40–45 kDa for FcγR isoforms (FcγRIa: rabbit polyclonal anti-CD64, Santa Cruz Biotechnology sc-15364, RRID AB_2103451, 10 μg/ml; FcγRIIIa: rabbit monoclonal anti-CD16, Abcam ab109223, RRID AB_10863447, dilution 0.5 μg/ml), 37 kDa for GAPDH (rabbit monoclonal anti-GAPDH, Cell Signaling Technology 2118, RRID AB_561053, dilution 1:6000) and 55 kDa for βIII-tubulin (mouse monoclonal anti-β-III-tubulin, Millipore MAB1637, RRID AB_2210524, dilution 1:6000). GAPDH (37 kDa) was used as a loading control for the western blots of FcγRIa (45–50 kDa); βΙΙΙ-tubulin (55 kDa), for western blots of FcγRIIIa (37 kDa). Since all tissues express FcγR, and we could not locate an FcγR knockout mouse, loading buffer was used as a negative control; lysates of peritoneal macrophages (30 μg per lane) were used as a positive control. Densitometric analysis was performed using Image Studio Lite software (RRID SCR_014211), v4.0 (Licor Biotechnology). Densitometric values for target proteins were normalized to densitometric values of loading controls (GAPDH for FcγRIa and βIII-tubulin for FcγRIIb, FcγRIIIa, and FcRn) in the same sample. FcγR protein expression was investigated in 2 cultures from each of 2 independent dissections.

### Flow cytometry

Rat cortical cells were dissociated, suspended in phosphate-buffered saline (PBS) containing 2% fetal bovine serum (FBS, LifeLine Cell Technology), and divided into flow cytometry tubes (5 ml polystyrene round-bottom tubes) for labeling, with 5 × 10^6^ cells/ml/tube. Cells were pelleted by centrifugation at 250×g for 5 min and re-suspended in primary antibody solutions: anti-FcγRI (rabbit polyclonal anti-CD64, Santa Cruz Biotechnology sc-15364, RRID AB_2103451, 2 μg/ml) and anti-FcγRII (mouse anti-rat CD32, BD Biosciences 550271, RRID AB_393568, 2 μg/ml) in PBS with 2% FBS. Cells were incubated for 40 min at room temperature on an orbital shaker. Cells were then centrifuged and washed with PBS, fixed for 30 min with 2% paraformaldehyde (PFA, Sigma), and permeabilized with 0.25% Triton X-100 (Fisher Scientific) in PBS for 10 min. After permeabilization, cells were centrifuged and incubated with primary antibodies against MAP2b (guinea pig polyclonal anti-MAP2b, Synaptic Systems 188,004, RRID AB_2138181, dilution 1:1000) or GFAP (guinea pig polyclonal anti-GFAP, Synaptic Systems 173,004, RRID AB 10641162, dilution 1:1000) in PBS containing 1% bovine serum albumin (BSA) and 10% goat serum for 1 h at room temperature. Cells were washed twice by adding PBS to each tube, shaking vigorously and then centrifuging for 5 min before re-suspending in PBS. Cells were incubated with fluorescently labeled secondary antibodies in PBS for 45 min at room temperature. Phycoerythrin (PE)-labeled secondary antibody (PE-conjugated donkey anti-guinea pig F(ab’)2, Jackson ImmunoResearch Labs 706-116-148, RRID AB_2340455, dilution 1:1000) was used to detect MAP2b- and GFAP-labeled cells. AlexaFluo-488 goat anti-mouse F(ab’)2 antibody was used to detect FcγRII (CD32)-labeled cells (Jackson ImmunoResearch Labs 115-546-006, RRID AB_2338860, dilution 1:1000), while AlexaFluor 647 goat anti-rabbit IgG (H+L) was used to detect FcγRI (CD64)-labeled cells (Thermo Fisher Scientific A-21245, RRID AB_2535813, dilution 1:1000). After incubation with secondary antibodies, the cells were washed twice with PBS and kept at 4 °C until analysis. A subset of cells were fixed and incubated without antibodies as a negative control. Additional controls included cells incubated with one primary antibody at a time, and cells incubated only with secondary antibodies alone and in combination, in order to set the maximum threshold for non-specific binding. A FACS Calibur (BD Biosciences) instrument was used for acquisition and compensation at the UC Davis Flow Cytometry Shared Resource. Compensation was performed using the data from cells incubated with each of the cellular markers individually. Data were analyzed and plotted using FlowJo V10 (RRID SCR_008520, FlowJo, LLC, Ashland, OR).

### Immunocytochemistry

For cell surface staining, cortical and hippocampal cell cultures were grown on glass coverslips (5–9 × 10^4^ cells/cm^2^) and fixed on DIV 7 in 4% PFA for 20 min at room temperature. Cells were blocked with PBS containing 1% BSA and 10% goat normal serum (Vector Laboratories) for 1 h at room temperature. After blocking, cultures were stained with primary antibodies specific for FcγRI (rabbit polyclonal anti-CD64, Santa Cruz Biotechnology sc-15364, RRID AB_2103451, 2 μg/ml) or FcγRII (mouse anti-rat CD32, BD Biosciences 550271, RRID AB_393568, 2 μg/ml) in PBS with 2% FBS overnight at room temperature. Cultures were then rinsed with PBS, permeabilized with 0.25% Triton X-100 in PBS for 5 min and stained with antibodies specific for GFAP (guinea pig polyclonal anti-GFAP, Synaptic Systems 173 004, RRID AB_10641162, dilution 1:1000) and MAP2b (guinea pig polyclonal anti-MAP2b, Synaptic Systems 188 004, RRID AB_2138181, dilution 1:1000) for 1 h at room temperature. After incubation with primary antibodies, cultures were rinsed 3 times with PBS and then incubated with secondary antibodies (AlexaFluor-568 goat anti-guinea pig IgG, Thermo Fisher Scientific A-11075, RRID AB_141954; AlexaFluor-647 goat anti-rabbit IgG, Thermo Fisher Scientific A-21245, RRID AB_2535813; and AlexaFluor-488 goat anti-mouse F(ab’)2, Jackson ImmunoResearch Labs 115-546-006, RRID AB_2338860, all diluted 1:1000) for 30 min. Cells were then rinsed twice with PBS, quickly rinsed in water, and mounted on slides in Prolong Gold Antifade with DAPI (Molecular Probes, Thermo Fisher Scientific). Images were acquired using a 40× objective on the ImageXpress MicroXL high-content screening system (Molecular Devices).

### Proteotype analysis

Cell surface capture (CSC) technology was used as previously described [53] to identify the cell surface proteotype (e.g., surfaceome) in DIV 7, DIV 14, and DIV 21 hippocampal cell cultures (seeded at a density of 1 × 10^5^ cells/cm^2^). Briefly, surface glycoproteins were gently oxidized with 2 mM NaIO4 and subsequently biotinylated with 5 mM biocytin hydrazide for 20 min. Cells were harvested by scraping and lysed in lysis buffer (50 mM ammonium bicarbonate, 0.25% Rapigest, 10 mM TCEP) by sonication using a VialTweeter (Hielscher Ultrasonics Gmbh, Germany). Proteins were alkylated for 30 min with 10 mM iodoacetamide (room temperature in the dark) and digested with trypsin overnight at 37 °C, in an enzyme-to-protein ratio of 1:50. In order to inactivate trypsin, samples were boiled 20 min and subsequently incubated with 1 mM PMSF for 15 min at 37 °C while shaking (300 rpm) and then acidified with 10% formic acid to pH 3. Samples were centrifuged (10 min, 16,000 × g) before capturing biotinylated glycopeptides using streptavidin affinity matrix (Pierce, Ultralink). Glycopeptides captured on the streptavidin affinity matrix were washed as previously described and incubated with PNGase F at 37 °C for 18 h to release N-glycosylated peptides. Samples were acidified to pH 2–3 with formic acid, and peptides were desalted with C18 UltraMicroSpin columns (The Nest Group) according to the manufacturer’s instruction and dried in a SpeedVac concentrator (Thermo Scientific, Rockford, USA).

For mass spectrometric (MS) analysis, peptides were reconstituted in 3% acetonitrile and 0.1% formic acid, and separated by reverse-phase chromatography on a high-pressure liquid chromatography (HPLC) column (75-μm inner diameter; New Objective) packed in-house with a 15-cm stationary phase (Magic C18AQ, 200 Å, 1.9; Michrom Bioresources) and connected to an EASY-nLC 1000 instrument combined with an autosampler (Proxeon). The HPLC was coupled to a Q Exactive plus mass spectrometer equipped with a nanoelectrospray ion source (Thermo Scientific). Peptides were loaded onto the column with 100% buffer A (99% H2O, 0.1% formic acid) and eluted at a constant flow rate of 300 nL/min for 30 min with a linear gradient from 6 to 20% buffer B (99.9% acetonitrile, 0.1% formic acid), followed by 15 min from 20 to 35% buffer B and a washing step with 90% buffer B. Mass spectra were acquired in a data-dependent manner (top 15). In a standard method for medium-to-low abundant samples, high-resolution MS1 spectra were acquired at 70,000 resolution (automatic gain control target value 3 × 10^6^) to monitor peptide ions in the mass range of 375–1500 m/z, followed by high-energy collisional dissociation (HCD)-MS/MS scans @ 35,000 resolution (automatic gain control target value 1 × 10^6^). To avoid multiple scans of dominant ions, the precursor ion masses of scanned ions are dynamically excluded from MS/MS analysis for 30 s. Single-charged ions and ions with unassigned charge states or charge states above 6 were excluded from MS/MS fragmentation.

For data analysis, SEQUEST software v27.0 was used to search fragment ion spectra for a match to tryptic peptides with maximally two missed cleavage sites from a protein database, which was composed of the *Rattus norvegicus* reference proteome (Uniprot, organism ID 10116), various common contaminants and sequence-reversed decoy proteins. The precursor ion mass tolerance was set to 20 ppm. Carbamidomethylation was set as a fixed modification on all cysteines. The PeptideProphet and the ProteinProphet tools of the trans-proteomic pipeline (TPP v4.6.2) were used for the probability scoring of peptide spectrum matches, and inferring protein identifications were filtered to reach an estimated false-discovery rate of ≤ 1%. Finally, proteins were filtered for cell surface glycoproteins as previously described [53]. In brief, each identified protein was required to be identified with at least one peptide containing the consensus NXS/T glycosylation motif containing an asparagine to aspartic acid deamidation site with a MS measured mass difference of 0.986 Da, indicating the cell surface labeling and the enzymatic deamidation during the CSC workflow.

### Cross-linking studies with IgG immune complex (IgG-IC)

To form an antibody-antigen immune complex, normal mouse IgG and affinity-purified rat anti-mouse IgG with minimal cross-reactivity to rat serum proteins were used as previously described [54]. The mouse IgG was dialyzed overnight against sterile PBS using a 10,000 Da molecular weight cutoff Slide-A-Lyzer MINI dialysis device (Thermo Fisher Scientific) to remove sodium azide. The dialysate was spin-concentrated using Amicon ultra centrifugal filter units (Millipore) before complexing. The IgG-IC was prepared fresh before each experiment by incubating mouse IgG with rat anti-mouse IgG in a 1:1 ratio (*w*/*w*) for 1 h at room temperature prior to addition to the cells, with the exception of the internalization studies, where incubation of cultures with each component of the immune complex (AlexaFluor 647-labeled rat anti-mouse IgG, Jackson ImmunoResearch Labs 415-605-166, RRID AB_2340285; and normal mouse IgG, Santa Cruz Biotechnology sc-2025, RRID AB_737182, concentration ratio 1:1) was performed sequentially. Cultures treated with the individual components of the IgG-IC were used as controls.

### Calcium imaging

To measure intracellular calcium concentration ([Ca^2+^]_i_), dissociated hippocampal neurons were seeded at a density of 1.5–3.0 × 10^4^ cells/cm^2^ (1–3 wells per condition in 4 independent dissections) and cultured on Costar 96-well plates (Corning). On DIV 7, cells were loaded with the Ca^2+^-sensitive dye, Fluo-4 AM (2 μM; Thermo Fisher Scientific), in imaging buffer (10 mM HEPES 140, pH 7.4, mM NaCl, 5 mM KCl, 2 mM MgCl_2_, 2 mM CaCl_2_, and 10 mM glucose) for 30 min in a 5% CO_2_ humidified incubator. Cultures were then washed with imaging buffer and transferred onto the stage of the ImageXpress MicroXL high-content imaging system. IgG-IC was applied to the cells, and full-frame FITC images were captured in one randomly chosen field per treatment (2 frames/s for 2.0–2.2 min) using MetaXpress software (Molecular Devices). Image capture was initiated at *t* = 0 s; a half volume of IgG-IC or of a rat anti-mouse IgG control or a mouse IgG control was added to an equal volume of imaging media at *t* = 10 s (20th frame); KCl (50 mM) was added at the end of each experiment (*t* = 110 s) to verify cell viability. A total of 240–260 images were acquired per well, and 1–3 wells were used per treatment in cultures from 4 independent dissections. Images were acquired in sequential mode between individual wells so that each well would receive the respective treatment (vehicle or IgG-IC) and images would be acquired from that well before proceeding to treatment of the next well. In instances where more than one well per condition were used, images from one well per condition across conditions were acquired as a first set, then the whole procedure was repeated for the next set of wells, in order to minimize as much as possible the influence of time between incremental exposures. Microscope stage coordinates were recorded for each chosen field.

To quantify [Ca^2+^]_i_, images were thresholded to remove background fluorescence and regions of interest (ROIs) were drawn around the soma of each Fluo-4-loaded cell. Fluorescence intensity values across all acquired images per ROI were logged into Excel spreadsheets (Microsoft Corporation). Custom Excel macros were written to automate data analysis. Briefly, ROIs with an area < 30 μm^2^ were excluded from analysis, and fluorescence data (F) for each of the remaining ROIs were normalized to its baseline fluorescence value (*F*_*0*_; frames 1–20). Only cells in which addition of KCl resulted in a spike in normalized fluorescence intensity with amplitude greater than 10% were included in further analyses. For each cell, the maximum fluorescence following application of IgG IC (*F*; measured in arbitrary fluorescence units) was normalized to the baseline fluorescence (*F*_*0*_) for that cell and expressed as (*F* − *F*_0_)/*F*_0_ or Δ*F*/*F*_0_. Cells were considered responsive to treatment when the peak change in fluorescence intensity (Δ*F*/*F*_*0*_) after treatment was > 50% of baseline fluorescence. The total number of IgG-IC-responsive cells in 1–3 wells for each concentration of IgG-IC was divided by the total number of cells in those wells to derive the percentage of the cells responding. Data from IgG-exposed cells were compared to data from cells exposed to an equal volume of fresh imaging media.

### Erk phosphorylation assays

DIV 7 hippocampal cell cultures were treated with fresh culture media, IgG-IC at 10 or 100 μg/ml, or rat anti-mouse IgG at 10 or 100 μg/ml (1 culture per condition in each of 3 independent dissections). Following 30 min of exposure at 37 °C in a 5% CO_2_ humidified incubator, cultures were lysed with ice-cold lysis buffer (as described for western blotting). Protease and phosphatase inhibitors (1X Halt Protease and Phosphatase Inhibitor Cocktail; Thermo Fisher Scientific) were added to the lysis buffer immediately before cell lysis. Protein concentration was determined using the Pierce BCA Protein Assay Kit (Thermo Fisher Scientific), and 30 μg of protein from each sample was separated using 7.5 or 10% Mini-PROTEAN TGX precast gels (Bio-Rad), then transferred to PVDF membrane. Membranes were blocked with Odyssey Blocking Buffer (Licor Biotechnology) for 30 min at room temperature and then incubated overnight at 4 °C with primary antibodies against phospho-Erk1/2 (Phospho Thr 202 & Tyr204/rabbit monoclonal anti-phospho-p44/42 MAPK (Erk1/2), Cell Signaling Technology 9106, RRID AB_331768, dilution 1:1000) diluted in blocking buffer with 0.1% Tween-20. Antibodies against GAPDH were used as loading controls. After washing in PBS supplemented with 0.1% Tween-20, membranes were incubated with secondary antibodies diluted in blocking buffer with 0.1% Tween-20 and 0.01% SDS for 1 h at room temperature. After extensive rinsing, membranes were scanned using the Odyssey Infrared Imaging System (Licor Biotechnology). Blots were then incubated with stripping buffer (62.5 mM Tris-HCl, pH 6.8, with 2% SDS and 100 mM β-mercaptoethanol) for 1 h at 37 °C and then imaged to confirm the absence of bands. Stripped membranes were immunoblotted for total (phosphorylated and non-phosphorylated) Erk1/2 (Erk 1/2/mouse monoclonal anti-p44/42 MAP kinase, Cell Signaling Technology 4696, RRID AB_390780, dilution 1:2000). Antibodies against GAPDH (rabbit monoclonal anti-GAPDH, Cell Signaling Technology 2118, RRID AB_561053, dilution 1:6000) were used as loading controls. Following incubation with secondary antibodies, membranes were scanned using the Odyssey Infrared Imaging System (Licor Biotechnology). Secondary antibodies used for these experiments were IR700DX goat anti-mouse IgG (Rockland 610-130-121, RRID AB_220121), IR700DX goat anti-rabbit IgG (Rockland 611-130-122, RRID AB_220148), IR800 goat anti-mouse IgG (Rockland 610-132-121, RRID: AB220125) and IR800 goat anti-rabbit IgG (Rockland 611-132-122, RRID AB_220152), all diluted 1:10000. Densitometry values of bands, immunoreactive with antibody against total Erk1/2 (phosphorylated and non-phosphorylated) or antibody that specifically recognizes phosphorylated Erk, were normalized to densitometry values for GADPH-immunopositive bands in the same sample. Normalized data for treated samples were expressed as a percentage of normalized data for vehicle controls from the same experiment. As a positive control, lysates were collected from peritoneal macrophages (one culture per condition in a single experiment) exposed to 10 or 100 μg/ml IgG-IC for 5 min at 37 °C in a 5% CO_2_ humidified incubator.

### Internalization of immune complex in endosomes

To test whether binding of an immune complex (AlexaFluor 647-labeled rat anti-mouse IgG and normal mouse IgG) to FcγR leads to internalization of FcγR into endosomes, AlexaFluor 488-conjugated rat transferrin (Jackson ImmunoResearch Labs 012-540-050, RRID AB_2337161) was used to label the entire endocytic recycling pathway [55]. Briefly, hippocampal cell cultures (seeded at a density of 5 × 10^4^ cells/cm^2^) were exposed on DIV 7 to fresh growth media or 100 μg/ml of rat anti-mouse IgG for 10 min, followed by incubation with a mixture of 100 μg/ml of normal mouse IgG and 30 μg/ml labeled transferrin for 10 min. Incubations were performed at 37 °C in a 5% CO_2_ humidified incubator. Negative controls were incubated with rat anti-mouse IgG and transferrin, but without normal mouse IgG. In a subset of experiments, cultures were pre-incubated for 5 min with anti-FcγRI (Santa Cruz Biotechnology sc-15364, RRID AB_2103451) or anti-FcγRIIb (BD Biosciences 550271, RRID AB_393568) antibodies (at 10 μg/ml each) to block the FcγR.

Following the 10-min transferrin exposure, cells were fixed with 4% PFA. Images were acquired at × 40 magnification using the ImageXpress MicroXL high-content imaging system. Twenty-five sites were imaged per well in two wells per condition in each of 3 (without Fc block) or 5 (with Fc block) independent dissections. Images were analyzed using MetaXpress software. A custom data analysis module was created using Custom Module Editor to calculate the average number of rat anti-mouse fluorescent puncta co-localized with transferrin per cell.

### Statistical analysis

Graphs were constructed in Graphpad Prism (RRID SCR_002798; v5.01), and statistical analyses were performed using SPSS (RRID SCR_002865; version 22.0.0.1) unless otherwise specified. Statistical comparisons were performed using block design ANOVA (experimental days were used as blocks; a Tukey’s honest significant difference/HSD test was used to identify significant differences between groups) with the following exceptions: (i) comparisons of *Fcgr* transcript levels between hippocampal and cortical cell cultures and data from the transferrin endocytosis assays were analyzed by *t* test in Graphpad Prism; (ii) assessment of fold-change in *FcgR* mRNA expression post-exposure to IFNγ and of change in *FcgR* transcript expression in vivo were performed by built-in randomization algorithms in REST 2009 (separately for each litter), then fold-changes and 95% confidence intervals from each litter were copied into Graphpad Prism to calculate *p* values; and (iii) densitometric analysis of constitutive FcγR expression in cortical cells was performed in Graphpad Prism using two-way ANOVA.

## Results

### In vivo and in vitro expression of FcγR transcripts in the developing rat brain

**Fig. 1:**
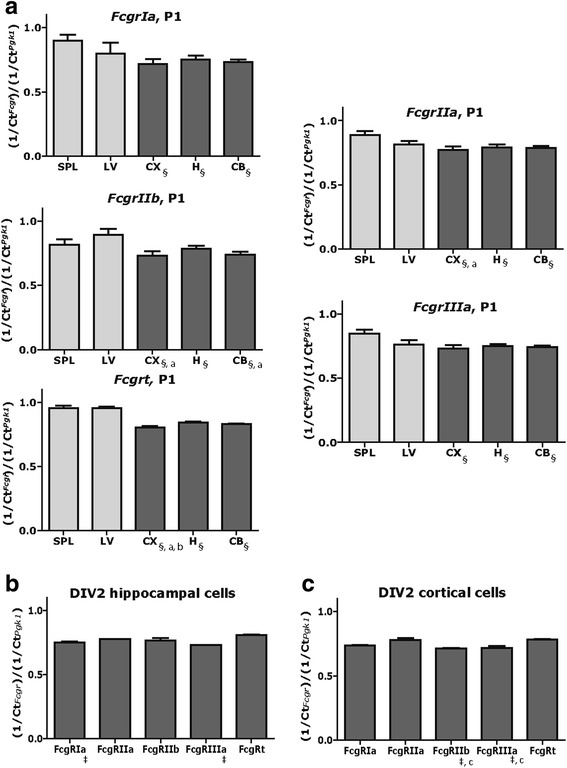
*Fcgr* are expressed in the developing rat brain and in primary neurons and astrocytes. **A** Gene expression in the developing brain at postnatal day 1 (P1). Results are presented as the inverse Ct value of the target gene normalized to the inverse Ct value for the reference gene (*Pgk1*) in the same sample. Data from two independent litters are presented as the mean ± SEM. Section sign indicates significantly lower compared to the spleen; a: significantly lower compared to hippocampus; b: significantly lower compared to cerebellum (block design ANOVA with Tukey’s HSD post hoc test). **B**, **C** In vitro gene expression in primary hippocampal (**B**) and cortical cell (**C**) cultures on the second day in vitro (DIV2). Data from two independent dissections are presented as the mean ± SEM. Double dagger indicates significantly lower compared to *Fcgrt*; c: significantly lower compared to *FcgrIIa*, as determined by block design ANOVA with Tukey’s HSD post hoc test

We first examined the transcript profile of FcγR in the developing rat brain. *FcgrIa*, *FcgrIIa*, *FcgrIIb*, *FcgrIIIa*, and F*cgrt* mRNA was quantified in the cortex, hippocampus, and cerebellum of P1 male and female pups using qPCR. The spleen and liver from these pups were used as control tissues. All five *Fcgr* transcripts were detected in all three brain regions and control tissues in both sexes (Fig. 1a). Significant differences in transcript levels between males and females were not detected by 2-way ANOVA (testing for sex and tissue effects; *p* set at 0.05) in any of the tissues examined; therefore, data from both sexes were pooled for this and all subsequent analyses. Significant differences were observed between different *FcgR* transcript levels within each of the different tissues (*F*(1,4)=116.880, *p* = 0.000 for spleen, *F*(1,4)= 103.749, *p* = 0.000 for liver, *F*(1,4) = 54.082, *p* = 0.000 for cortex, *F*(1,4)=57.298, *p* = 0.000 for hippocampus, *F*(1,4)= 74.946, *p* = 0.000 for cerebellum), and *Fcgrt* was the most abundant transcript across all tissues and brain regions tested (*p* = 0.000; block design ANOVA with Tukey’s post hoc test; *n* = 6 pups from each of 2 independent litters). Transcript levels for each *FcgR* differed between tissues (*F*(1,4) = 52.436, *p* = 0.000 for *FcgRIa*, *F*(1,4) = 132.459, *p* = 0.000 for *FcgRIIa*, *F*(1,4) = 161.012, *p* = 0.000 for *FcgRIIb*, *F*(1,4) = 72.733, *p* = 0.000 for *FcgRIIIa*, *F*(1,4) = 254.119, *p* = 0.000 for *FcgRt*; block design ANOVA). All *Fcgr* transcripts were expressed at lower levels in the cortex, hippocampus, and cerebellum compared to the spleen (*p* = 0.000; Tukey’s HSD post hoc test). *Fcgr* levels also differed between brain regions. Specifically, the cortex expressed less *FcgrIIa* (*p* = 0.006) and *FcgrIIb* (*p* = 0.000) compared to the hippocampus, and cortical levels of *Fcgrt* were lower than in the hippocampus (*p* = 0.000) or cerebellum (*p* = 0.001). *FcgrIIb* was less abundant in the cerebellum than in the hippocampus (*p* = 0.000). No differences in *FcgrIa* or *FcgrIIIa* expression were observed between brain regions.

To assess whether *Fcgr* transcript levels changed as a function of age, *Fcgr* transcript levels in tissues from P7 pups (*n* = 11 pups from two litters, except for the spleen where *n* = 8 from two litters) were compared to levels quantified in P1 tissues (*n* = 12 pups from two litters). *Fcgr* transcript levels were generally increased in the brain of P7 pups relative to their P1 littermates (Additional file 1). Significant changes were observed in the cerebellum where *FcgrIIb* (2.661 fold; 95% CI, 0.513–23.645; *p* = 0.004) and *Fcgrt* (2.294 fold; 95% CI, 0.679–9.559; *p* = 0.001) increased from P1 to P7, and in the cortex, where *FcgrIIb* (2.491 fold; 95% CI, 0.343–22.422; *p* = 0.034) increased from P1 to P7. Larger confidence intervals for *FcgrIIb* and *Fcgrt* in the cerebellum are due to the variability in the Ct values obtained between different animals and could be due to regional differences in FcγR expression between different areas of the cerebellum (although the entire cerebellum was removed and stored in RNALater across all animals, the pieces that were used for analysis were randomly chosen). In contrast to the cerebellum and the cortex, in the spleen, *FcgrIa* (0.276 fold; 95% CI, 0.034–8.977; *p* = 0.018), *FcgrIIa* (0.244 fold; 95% CI, 0.077–3.074; *p* = 0.001), and *Fcgrt* (0.452 fold; 95% CI, 0.130–3.412; *p* = 0.009) decreased significantly from P1 to P7. *Fcgr* transcript levels did not change significantly between P1 and P7 in the hippocampus or liver. Results for liver and spleen can be found in Additional file 2.

To determine whether *FcgrIa*, *FcgrIIa*, *FcgrIIb*, *FcgrIIIa*, and *Fcgrt* transcripts are also expressed in the developing rat brain in vitro, and particularly in non-immune cells, mRNA levels of these *Fcgr* genes were quantified by qPCR in primary hippocampal and cortical cells dissociated from P1 rats (pooled male and female brain regions). These cultures were comprised of MAP2b-positive neurons and GFAP-positive astrocytes and were confirmed to be devoid of microglia and endothelial cells, as determined by lack of immunolabeling for CD68 and Iba-1 (microglial markers) and CD31 (endothelial cell marker), and to contain < 1% CD11b immunopositive cells (Additional files 3 and 4). Consistent with in vivo expression patterns, on DIV 2, all five *Fcgr* transcripts were detected in hippocampal (Fig. 1b) and cortical cell cultures (Fig. 1c). There were significant differences in expression levels between different *Fcgr* transcripts in hippocampal cell cultures (*F*(1,4) = 12.379, *p* = 0.016, block design ANOVA; *n* = 3 cultures per group in each of two dissections). As observed in vivo, *Fcgrt* was expressed at significantly higher levels than either *FcgrIa* (0.060; *p* = 0.034) or *FcgrIIIa* (0.079; *p* = 0.013) (Fig. 1b). Small differences between transcript levels were also observed in DIV 2 cortical cell cultures (Fig. 1c; *F*(1,4) = 14.087, *p* = 0.013, block design ANOVA), with *Fcgrt* and *FcgrIIa* being more abundantly expressed than *FcgrIIb* (mean difference: 0.070; *p* = 0.023 and 0.066; *p* = 0.028, respectively) or *FcgrIIIa* (0.065; *p* = 0.029 and 0.061; *p* = 0.036, respectively). Comparison of each *Fcgr* transcript between the two culture types (*t* test) revealed a significant difference only for *Fcgrt*, which was slightly more abundant in hippocampal cell cultures compared to cortical cell cultures (mean difference = 0.025; *p* = 0.026; df = 2).

To further assess the ontogeny of *Fcgr* mRNA expression in vitro, *Fcgr* transcript levels were determined from total RNA isolated from primary hippocampal and cortical cell cultures on DIV 7, 14, and 21 and compared to levels on DIV 2. Significant changes in *Fcgr* expression as a function of DIV were observed in both culture types. The relative expression values along with 95% confidence intervals and *p* values for each gene in hippocampal and cortical cell cultures are listed in Table 2 (pooled data from 6 cultures derived from 2 independent dissections; REST2009 software built-in statistical analysis). In hippocampal cell cultures, *FcgrIa*, *FgcrIIa*, *FcgrIIb*, and *FcgrIIIa* transcript levels were decreased on DIV 7, 14, and 21 compared to DIV 2, while *Fcgrt* expression remained constant across all DIV. In cortical cell cultures, *FcgrIa*, *FcgrIIa*, *FcgrIIb*, and *FcgrIIIa* levels were lower on DIV 7 compared to DIV 2, while only *FcgrIIa* and *FcgrIIIa* levels were reduced on DIV 14 and DIV 21 compared to DIV 2. *Fcgrt* levels were significantly increased on DIV 14 but not changed on DIV7 or DIV21 compared to DIV 2.

**Table 2:** Changes in *Fcgr* expression on DIV 7, 14, and 21 relative to DIV 2

	DIV 7 vs. DIV 2			DIV 14 vs. DIV 2			DIV 21 vs. DIV 2		
Gene	R.Exp.	95% CI	*p*	R.Exp.	95% CI	*p*	R.Exp.	95% CI	*p*
Hippocampal cells									
*FcgrIa*	0.084	0.016–0.620	0.002↓	0.021	0.003–0.241	0.000↓	0.036	0.005–1.432	0.002↓
*FcgrIIa*	0.275	0.088–1.005	0.002↓	0.183	0.022–0.661	0.001↓	0.240	0.048–2.567	0.013↓
*FcgrIIb*	0.427	0.117–2.224	0.045↓	0.327	0.043–3.014	0.058	0.215	0.036–1.453	0.007↓
*FcgrIIIa*	0.112	0.011–1.043	0.007↓	0.061	0.011–0.468	0.001↓	0.064	0.011–0.906	0.001↓
*Fcgrt*	1.209	0.406–3.412	0.512	1.202	0.461–3.745	0.473	1.105	0.320–3.341	0.737
Cortical cells									
*FcgrIa*	0.264	0.046–1.625	0.016↓	0.110	0.007–9.227	0.080	0.051	0.003–9.892	0.074
*FcgrIIa*	0.392	0.080–1.059	0.005↓	0.262	0.075–0.835	0.001↓	0.093	0.034–0.259	0.002↓
*FcgrIIb*	0.386	0.106–1.077	0.010↓	0.527	0.037–36.8	0.535	0.308	0.025–5.478	0.185
*FcgrIIIa*	0.293	0.063–1.359	0.009↓	0.039	0.009–0.147	0.001↓	0.008	0.002–0.024	0.001↓
*Fcgrt*	2.543	0.532–9.585	0.061	2.135	1.101–4.622	0.002↑	0.991	0.491–2.124	0.967

Relative expression = (concentration of Fcgr gene)/(concentration of reference gene Pgk1) [48]. Wherever the change in expression between two DIVs is statistically significant (*p* value < 0.05; as determined by built-in randomization techniques in REST 2009 analysis software), arrows represent direction of change in expression (↑ up; ↓ down)

R. Exp. relative expression, CI confidence interval

### FcγRIa, FcγRIIb, and FcγRIIIa proteins are expressed in neurons and astrocytes

**Fig. 2:**
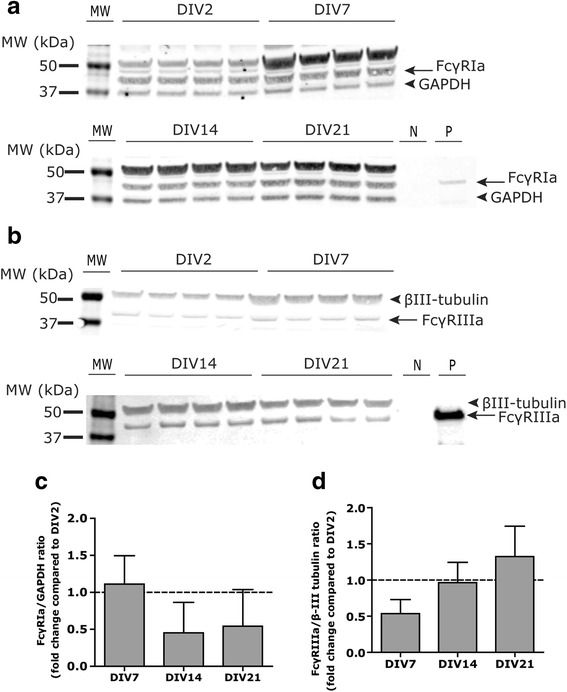
Western blot analyses of FcγRIa and FcγRIIIa expression in cortical cell cultures. Representative blots showing FcγRIa (**a**) and FcγRIIIa (**b**) expression in cortical cells on DIV 2, 7, 14, and 21. Each sample was also probed for either GAPDH (FcγRIa immunoblots) or βIII-tubulin (FcγRIIIa immunoblots) as loading controls. *MW* molecular weight standards, *N* lysis buffer used as a negative control, *P* lysates from peritoneal macrophages used as a positive control. Densitometric analyses showed no significant effects of culture age on FcγRIa (**c**) or FcγRIIIa (**d**) expression. For each sample, the optical density of the FcγR immunopositive band was normalized to the optical density of the loading control. Data are presented as the fold-change (± SEM) in normalized optical density for each DIV relative to DIV 2 which is represented as the horizontal dotted line in the graphs

Having confirmed that non-immune cells in the developing rat brain express *Fcgr* and that the transcript profile of *Fcgr* in vitro resembles that in vivo, we next determined whether FcγR are expressed at the protein level in primary hippocampal and cortical cell cultures. We first used western blotting to interrogate FcγR protein expression in rat cortical cell cultures. Commercially available antibodies specific for FcγRIa (CD64; protein product of *FcgrIa*), FcγRIIb (CD32; protein product of *FcgrIIb*), FcγRIIIa (CD16; protein product of *FcgrIIIa*), and FcRn (protein product of *Fcgrt*) were initially tested against cell lysates of adult rat peritoneal macrophages as a positive control. Bands immunoreactive for FcγRIa and FcγRIIIa of the anticipated size (40–45 kDa) were detected in peritoneal macrophage lysates (not shown). In contrast, anti-FcγRIIb and anti-FcRn antibodies failed to react with macrophage cell lysates, suggesting that these antibodies are not suitable for western blotting. Similarly, FcγRIa (Fig. 2a) and FcγRIIIa (Fig. 2b), but not FcγRIIb or FcRn, were detected in cortical cell lysates collected on DIV 2, 7, 14, and 21. Densitometric analysis revealed no significant differences in FcγRIa or FcγRIIIa protein levels in cortical cells on DIV 7, DIV 14, and DIV 21 compared to DIV 2 (Fig. 2c, d; *F*(1,3) = 1.371, *p* = 0.4008 for FcγRIa; *F*(1,3) = 0.6472, *p* = 0.6353 for FcγRIIIa, 2-way ANOVA).

**Fig. 3:**
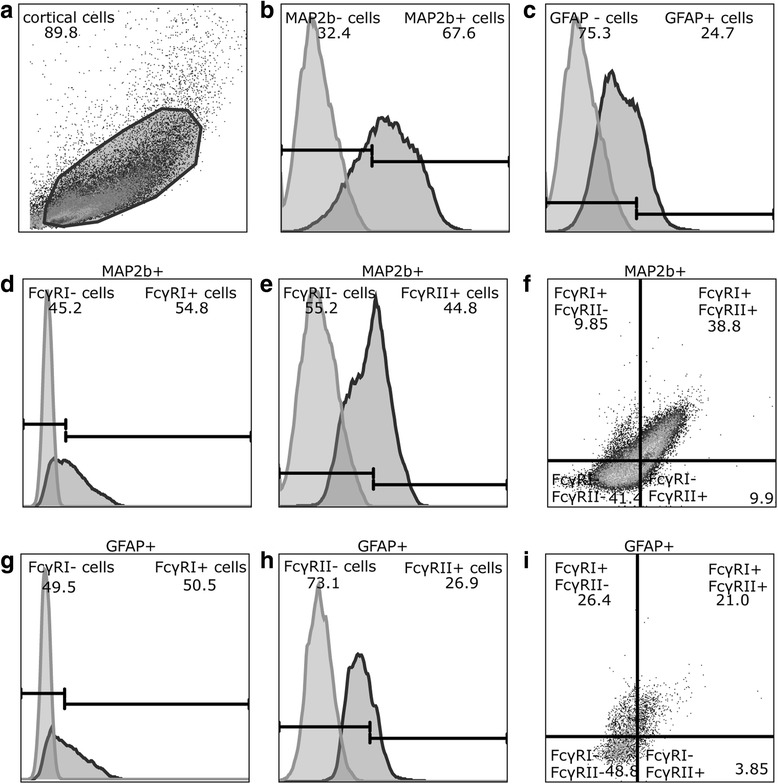
Cortical cells express FcγRIα and/or FcγRIIb receptors on their surface. Rat cortical cells (**a** side-scatter (*y*) versus forward-scatter (*x*) plot) were labeled for FcγRIa, FcγRIIb, and either MAP2b (**b**) or GFAP (**c**) (forward scatter (*y*) versus fluorescence intensity (*x*)). MAP2b-positive cells express either FcγRIa (**d**), or FcγRIIb (**e**) or both FcγRIa and FcγRIΙb (**f**). GFAP-positive cells express either FcγRIa (**g**), or FcγRIIb (**h**) or both receptors (**i**). Forward scatter (*y*) versus fluorescence intensity (*x*) were plotted for (**d**), (**e**), (**g**), and (**h**), whereas (**f**) and (**i**) were plotted as FcγRIIb (*x*) versus FcγRIa (*y*) fluorescence. The mean frequency of cells labeled with the respective markers is indicated in the graphs

In immune cells, the responses triggered as a result of immune complex recognition by FcγR can vary from cell to cell depending on the ratio of excitatory to inhibitory FcγR expressed on each cell [34, 35]. We hypothesized that FcγR subtype expression at the protein level similarly differs between individual neurons and/or astrocytes. Using flow cytometry of cells dissociated from the neocortex of P0 rat pups (Fig. 3a), we confirmed that cortical neurons, identified as MAP2b-immunopositive cells (Fig. 3b), and astrocytes, identified as GFAP-immunopositive cells (Fig. 3c), respectively, express FcγRIa (Fig. 3d, g) and FcγRIIb (Fig. 3e, h). Although some cortical neurons expressed either FcγRIa or FcγRIIb, a subset of cortical neurons expressed FcγRIa and FcγRIIb simultaneously at varying intensities (Fig. 3f). Similarly, a subset of cortical astrocytes was immunopositive for both FcγRIa and FcγRIIb (Fig. 3i).

**Fig. 4:**
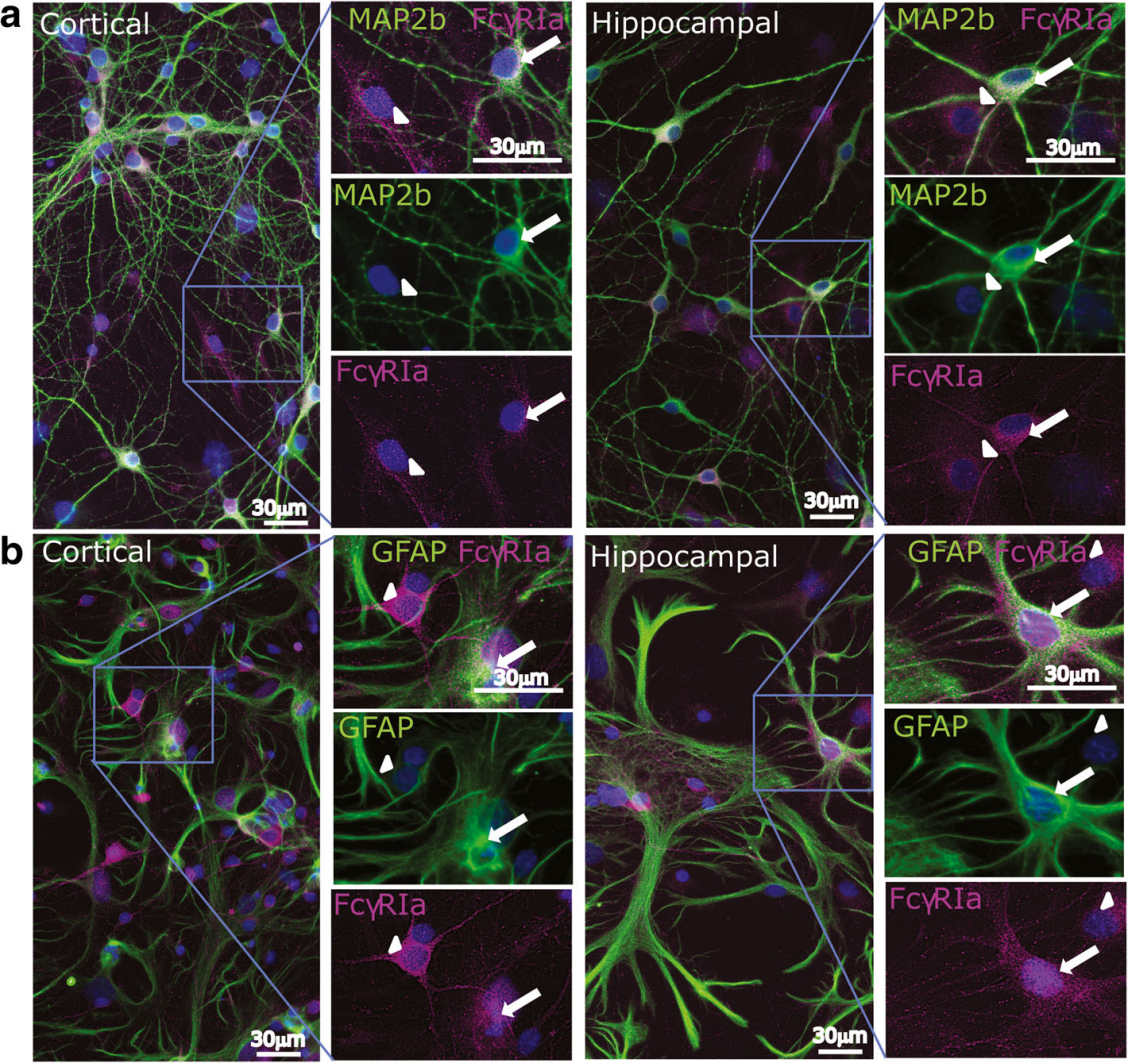
Cultured hippocampal and cortical neurons and astrocytes express FcγRIa on DIV 7. **a**, **b** Representative fluorescence micrographs and magnified inserts from DIV 7 cortical and hippocampal cell cultures immunolabeled for FcγRIa (magenta) and either MAP2b (**a** green**)** or GFAP (**b** green**)**. Nuclei are stained with Hoechst dye (blue). Arrows highlight co-labeling of cells with MAP2b or GFAP and FcγRIa. Arrowheads indicate immunolabeling for FcgRIa but not for MAP2b nor for GFAP

**Fig. 5:**
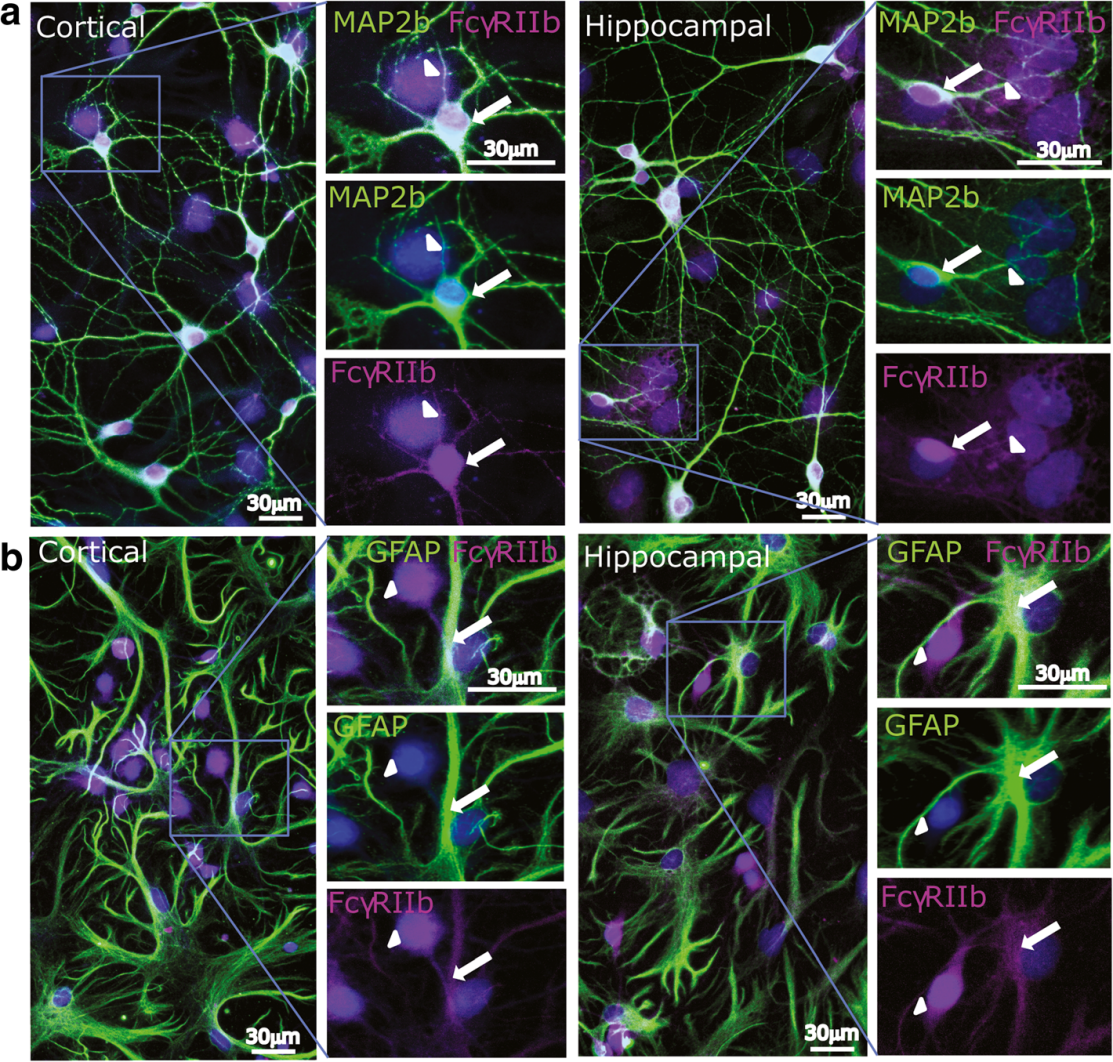
Cultured hippocampal and cortical neurons and astrocytes express FcγRIIb on DIV 7. **a**, **b** Representative fluorescence micrographs and magnified inserts from DIV 7 cortical and hippocampal cells immunolabeled for FcγRIIb (magenta) and either MAP2b (**a** green**)** or GFAP (**b** green**)**. Nuclei are stained with Hoechst dye (blue). Arrows highlight co-labeling of cells with MAP2b or GFAP and FcγRIIb. Arrowheads indicate immunolabeling for FcgRIIb but not for MAP2b nor for GFAP

To assess the subcellular expression of FcγR in neurons and astrocytes, we used immunocytochemistry to label hippocampal and cortical cells at DIV 7. Results confirmed our flow cytometry data, showing hippocampal and cortical neurons and astrocytes immunopositive for FcγRIa (Fig. 4) and for FcγRIIb (Fig. 5). Although MAP2b-immunopositive neurons were consistently immunoreactive for FcγRIa and/or FcγRIIb, immunolabeling of GFAP-immunopositive astrocytes for FcγR was highly variable between and within cultures. Some GFAP-immunopositive cells in our cultures did not react with either FcγRIa or FcγRIIb antibodies. Confocal microscopy confirmed that when cells were fixed and permeabilized before FcγR staining, they revealed intracellular (in some cases including intranuclear) staining for FcγRIa and FcγRIIb in addition to surface staining (not shown); this pattern of intracellular staining was not observed in cells which were immunolabeled before fixation. Intracellular immunolabeling for FcγR in fixed, permeabilized cells could be explained by the presence of intracellular pools of FcγR in neurons and astrocytes, available for translocation to the cell membrane or, conversely, reflect internalized receptors. The presence of neuronal surface receptors in intracellular pools [56, 57], as well as the presence of intracellular pools of FcγR in leukocytes [58], have been described before.

FcγRIIIa and FcRn immunoreactivities were not detected by immunocytochemistry in hippocampal or cortical cell cultures. Although cultures of adult rat peritoneal cells were immunoreactive for FcRn (data not shown), FcRn immunoreactivity was not observed in hippocampal and cortical cultures. The absence of FcγRIIIa immunostaining and the presence of FcγRIIb immunostaining in primary cortical and hippocampal neurons are in contrast to our western blot data, suggesting that the anti-FcγRIIIa and anti-FcγRIIb antibodies we used are not dual purpose antibodies that would be suitable for both western blotting and immunocytochemistry. This possibility was confirmed by observations that the anti-FcγRIIb antibody detected FcγRIIb immunoreactivity in peritoneal cells by immunocytochemistry but not by western blotting (data not shown).

**Fig. 6:**
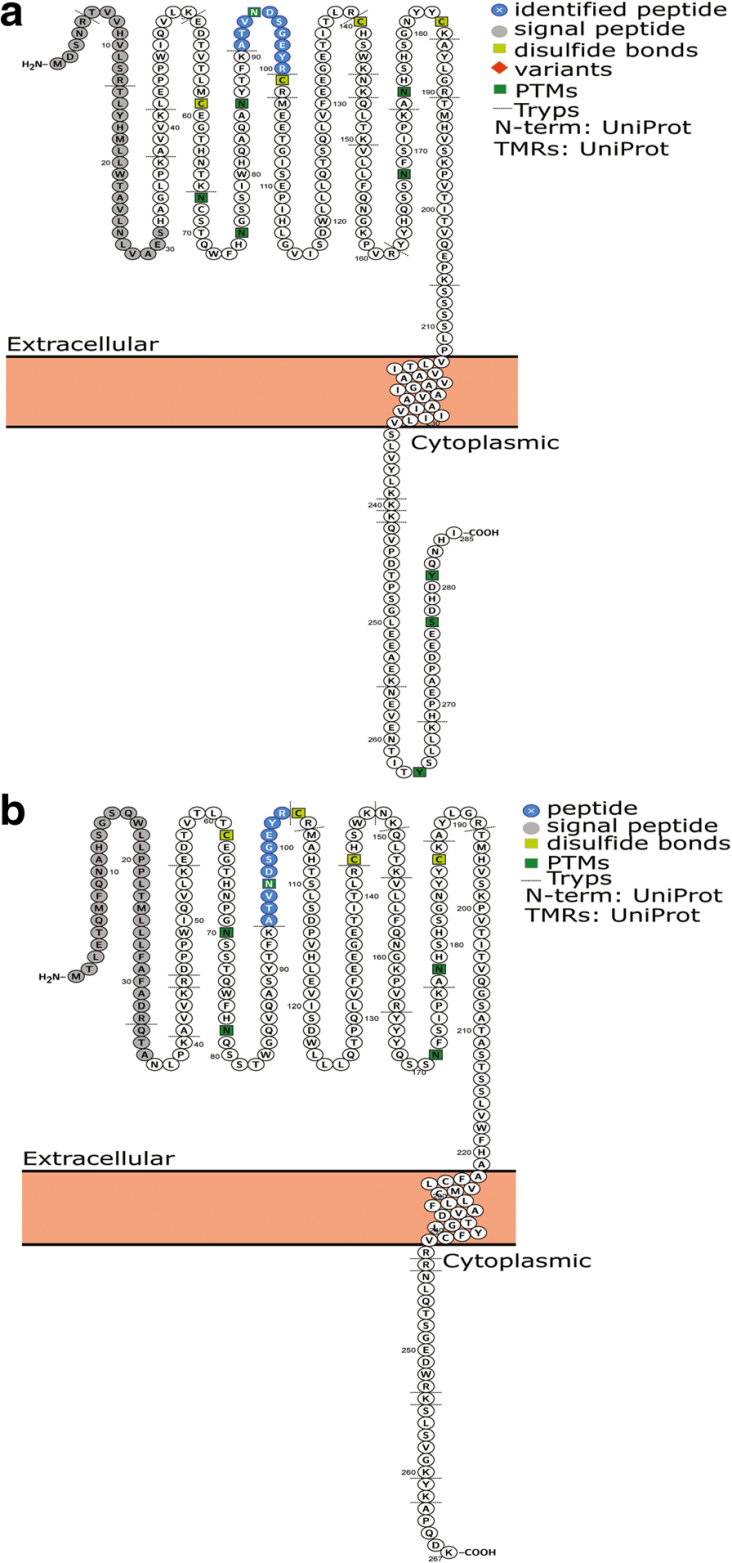
Cell surface capture proteomics identify FcγRIIb and FcγRIIIa in rat hippocampal neurons and astrocytes. **a**, **b** Sequence coverage of identified FcγR on topological prediction maps for rat FcγRIIb (**a** Uniprot ID Q63203**)** and rat FcγRIIIa (**b** Uniprot ID P27645**)** were generated using Protter (http://wlab.ethz.ch/protter). The sequence of the identified peptide is depicted in blue

To confirm surface expression of FcγR in cultures of neurons and astrocytes as detected by immunocytochemistry, we employed chemoproteomic cell surface capture (CSC) technology [53]. CSC technology enables surfaceome analysis and phenotyping of cells without antibodies by using a mass spectrometry-based strategy. Following selective cell surface labeling using a biotinylated probe, formerly N-glycosylated and cell surface-residing glycoproteins were identified. A non-proteotypic glycopeptide derived from either FcγRII or FcγRIII was identified in surfaceome preparations from hippocampal cell cultures at DIV 7, DIV 14, and DIV 21. Using the open source tool, Protter [59] (Fig. 6), the identified glycopeptide (ATVNDSGEYR) was shown to map to the extracellular domain of FcγRII (Uniprot ID Q63203; Fig. 6a) and FcγRIII (Uniprot ID P27645; Fig. 6b). These data provide additional and independent confirmation that the FcγR protein (FcγRII and/or FcγRIII) is located on the cell surface in hippocampal neuron-glia co-cultures.

### Factors that modulate FcγR expression and signaling in immune cells exert similar effects in primary hippocampal and cortical cell cultures

**Fig. 7:**
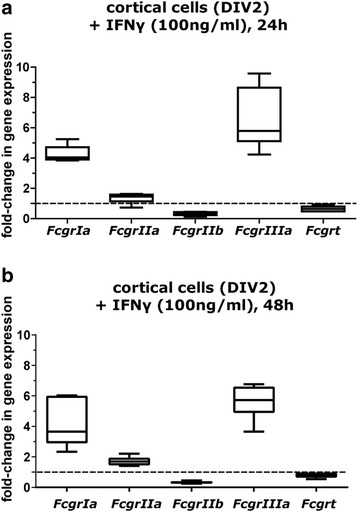
*Fcgr* mRNA levels in rat cortical cells are modulated by IFNγ. Effect of IFNγ (100 ng/ml) treatment for 24 h (**a**) or 48 h (**b**) on *Fcgr* expression in DIV 2 cortical cell cultures. The fold-change in *Fcgr* expression in IFNγ-treated cells relative to untreated cells (horizontal dotted line along *x*-axis) is plotted as whisker box plots. The horizontal line in each box represents the mean; lower and upper box limits, the 25th and 75th percentile, respectively; whiskers, the 1–99th percentile

IFNγ is an important inflammatory mediator produced by T cells and natural killer (NK) cells [60] that regulates FcγR expression in immune cells [61–64]. IFNγ has been suggested to upregulate FcγR expression in adult rat microglia at the protein level [65], but whether IFNγ alters expression of *Fcgr* mRNA in other cell types of the rodent brain is not known. To address this question, DIV 2 rat cortical cell cultures were exposed to recombinant rat IFNγ at 100 ng/ml, a concentration previously shown to influence signaling in cultured cortical neurons without affecting cell viability [66]. As determined using the statistical analysis package in the REST2009 software (that includes built-in randomization algorithms) to assess qPCR data, a 24-h exposure of cortical cell cultures to IFNγ significantly increased levels of *FcgrIa* (4.271-fold; 95% CI 3.705–4.837), *FcgrIIa* (1.351-fold; 95% CI 1.008–1.695), and *FcgrIIIa* (6.525-fold; 95% CI 4.403–8.646) but decreased levels of *FcgrIIb* (0.326-fold; 95% CI 0.201–0.451) and *Fcgrt* (0.653-fold; 95% CI 0.455–0.850) relative to sister cultures treated with vehicle (0.1% DMSO) (Fig. 7a). After 48 h of exposure to IFNγ (Fig. 7b), expression of *FcgrIa* (4.297-fold; 95% CI 2.247–6.348), *FcgrIIa* (1.723-fold; 95% CI 1.397–2.049), and *FcgrIIIa* (5.624-fold; 95% CI 4.444–6.803) remained significantly elevated, and levels of *Fcgrt* (0.802-fold; 95% CI 0.614–0.990) were still significantly decreased. Grubb’s test identified one of the 6 independent experiments as an outlier for *FcgrIIb* transcript levels after a 48-h exposure to IFN; with this outlier removed, FcgRIIb transcript levels were confirmed to be significantly decreased, similarly to the 24 h exposure to IFNγ (0.326-fold; 95% CI 0.2279–0.4245).

### Cross-linking of FcγR in primary neuron-glia co-cultures triggers downstream events observed following FcγR cross-linking in immune cells

In myeloid and lymphoid cells, cross-linking of excitatory FcγR, such as FcγRIa and FcγRIIIa, by IgG immune complexes (IgG-IC) triggers a rapid increase in intracellular calcium ([Ca^2+^]_i_) levels [32]. In contrast, mobilization of intracellular calcium is not observed upon ligation of inhibitory FcγR, such as FcγRIIb [32]. It has been suggested that IgG-mediated activation of FcγR increases [Ca^2+^]_i_ in peripheral neurons [67, 68]. We adapted a previously published approach of bath application of IgG-IC to stimulate [Ca^2+^]_i_ in hippocampal cell cultures [54]. To obtain high-quality images of individual cells and to distinguish between IgG-mediated effects on [Ca^2+^]_i_ and spontaneous, synchronized calcium oscillations normally occurring in mature hippocampal neurons in culture [69, 70], calcium imaging studies were performed on low density DIV 7 hippocampal cell cultures pre-loaded with the calcium indicator dye Fluo4. IgG-IC consisting of pre-complexed mouse IgG and rat anti-mouse IgG was added to the culture medium at varying concentrations.

**Fig. 8:**
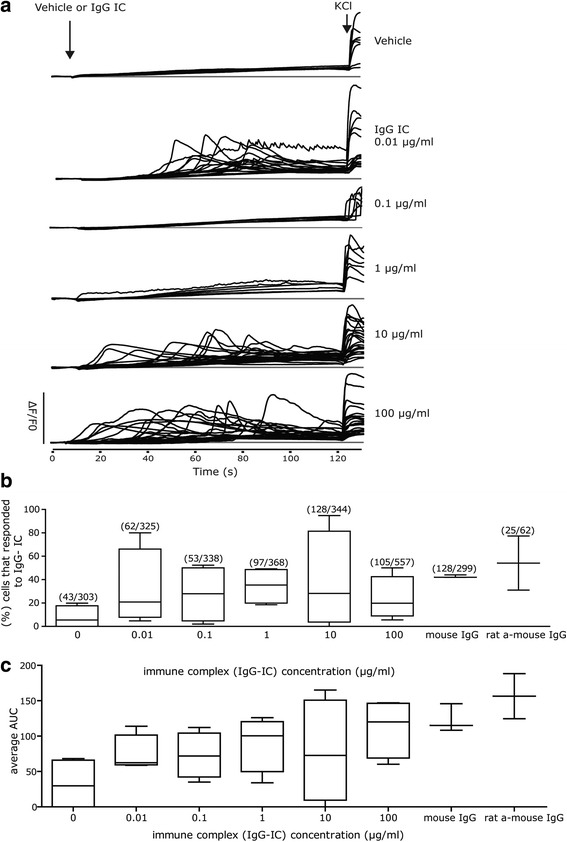
IgG-immune complex (IC) concentration-dependently increases [Ca^2+^]_i_ in primary hippocampal cells. DIV 7 hippocampal cell cultures were loaded with calcium indicator dye Fluo-4 prior to experimental manipulation. IgG-IC or vehicle were added 10 s after recordings started, and KCl (50 mM) was added at the end of each 2 min recording to verify cell viability. Changes in [Ca^2+^]_i_ were calculated by measuring changes in fluorescence intensity (ΔF/F_0_). **a** Randomly selected traces of [Ca^2+^]_i_ in KCl-responsive cells exposed to varying concentrations of IgG-IC (1:1 ratio of pre-complexed mouse IgG and rat anti-mouse IgG). **b** The percentage of cells that responded to IgG-IC or to the individual components of the immune complex (as evidenced by an increase in fluorescence > 50% of baseline fluorescence). The total number of cells that responded to IgG-IC or to the individual components of the immune complex (each at 100 μg/ml), divided by the total number of cells analyzed are provided in parentheses for each experimental condition. No significant differences were observed between groups. **c** Mean change in [Ca^2+^]_i_ as determined by the average area under the curve (AUC) of the ΔF/F_0_ tracing for cells that responded to IgG-IC or to the individual components of the immune complex (each at 100 μg/ml). No significant differences were observed between groups. Whisker box plots depict 25th–75th percentile range of values (box edges), median (horizontal line in box), and 1st–99th percentile range of values (whiskers)

Calcium traces of individual cells showed that under the culture conditions used in these experiments, neurons did not exhibit synchronized calcium oscillations (Fig. 8a). Analysis of calcium traces of individual cells indicated that exposure to IgG-IC caused concentration-dependent increases in [Ca^2+^]_i_ (Fig. 8b, c). Although the shape of calcium traces in IgG-IC responsive cells varied between IgG-IC concentrations and between experiments for any experimental condition, including vehicle, they generally exhibited one of two general responses: (i) a spike in [Ca^2+^]_i_ or (ii) a gradual increase in [Ca^2+^]_i_ (Fig. 8a). Quantification of the percentage of cells that responded to IgG-IC revealed that median values increased in a concentration-dependent manner up to 1 μg/ml (Fig. 8b). Calculation of the average area under the curve (AUC) for calcium traces revealed a concentration-dependent increase up to 1 μg/ml IgG-IC (Fig. 8c), whereas a reduction in average AUC was observed with 10 μg/ml IgG-IC and an increase was observed with 100 μg/ml IgG-IG. However, no statistically significant differences between means were found for either endpoint (block design ANOVA with statistical significance set at *p* = 0.05; *n* = 1–3 cultures in each of 4 independent dissections). The individual components of the IgG-IC were used in control experiments. Specifically, cells were incubated with either 100 μg/ml mouse IgG or with 100 μg/ml rat anti-mouse IgG and the percentage of responding cells as well as the average AUC of the calcium traces were calculated (Fig. 8b, c). Both mouse IgG and rat anti-mouse IgG at a very high concentration of 100 μg/ml increased [Ca^2+^]_i_ in some cells (Fig. 8b). The median AUC for calcium traces in response to 100 μg/ml mouse IgG was lower compared to 100 μg/ml IgG-IC, whereas the median AUC of the calcium traces at 100 μg/ml rat anti-mouse IgG was higher compared to 100 μg/ml IgG-IC (Fig. 8c). However, these differences were not significantly different from untreated cells, as determined by block design ANOVA (statistical significance set at *p* = 0.050; *n* = 1–3 cultures in each of 3 independent dissections for mouse IgG alone and 2 independent dissections for rat anti-mouse IgG alone). Although we did not test the individual components of the IgG-IC at concentrations lower than 100 μg/ml, based on evidence that IgG alone (in the absence of immune complex) has been shown to act antagonistically on FcγR signaling in immune signaling, we would not expect to observe a robust calcium response at lower concentrations of the individual components of the immune complex [71, 72].

To exclude the possibility that these studies were confounded by varying percentage of astrocytes comprising the total cell population, cultures from 2 of the 4 independent dissections used for this study were immunostained for MAP2b and GFAP following completion of the calcium assay. Fluorescent images of the same fields in the cells that had been used for the calcium assay were acquired to compare calcium traces from neurons versus astrocytes. Analysis of calcium traces of neurons alone was conducted as above, and the results (not shown) were similar to those reported above, independent of cell type, e.g., the percentage of neurons responsive to IgG-IC was concentration dependent, but not significantly different from vehicle control.

**Fig. 9:**
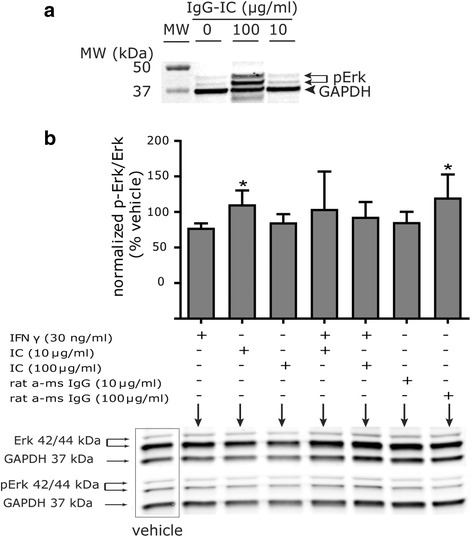
IgG-immune complex (IgG-IC) increases Erk phosphorylation in primary hippocampal cell cultures. **a** Rat peritoneal macrophages were exposed for 5 min to varying concentrations of IgG-IC, lysed, and immunoblotted for phosphorylated Erk (pErk). The optical density of pErk immunoreactive bands, normalized to GAPDH, were 600-fold higher in peritoneal cells exposed to 10 μg/ml IgG-IC and > 10,000-fold higher in peritoneal cells exposed to 100 μg/ml IgG-IC compared to cells exposed to media alone. **b** DIV 7 hippocampal cells were exposed for 30 min to 10 or 100 μg/ml IgG-IC (1:1 ratio of pre-complexed mouse IgG and rat anti-mouse IgG), or to 10 or 100 μg/ml rat anti-mouse IgG. Cell lysates were separated by SDS PAGE and then immunoblotted for pErk, total Erk, and GAPDH. The optical density of bands immunoreactive for pErk and total Erk was normalized to the optical density of GAPDH immunoreactive bands in the same sample. The ratio of pErk to Erk is plotted as a percentage of vehicle controls. Data are presented as the mean ± SEM. *MW* molecular weight standards

In immune cells, increased [Ca^2+^]_i_ as a result of cross-linking of excitatory FcγR has been linked to activation of the MAPK signaling pathway and Erk phosphorylation [32]. Similarly, we observed that IgG-IC increased levels of phosphorylated Erk (pErk) in adult rat peritoneal cells in a concentration-dependent manner (Fig. 9a). The ratio of pErk to Erk was increased significantly (60.44; *p* = 0.046; Tukey’s HSD) in DIV 7 hippocampal cell cultures exposed to IgG-IC at 10 μg/ml relative to control cultures treated with media alone but not at 100 μg/ml (Fig. 9b; (*F*(2,7) = 3.331, *p* = 0.026, block design ANOVA)). In contrast, when cells were exposed to rat anti-mouse IgG alone, 10 μg/ml had no effect on Erk phosphorylation whereas 100 μg/ml significantly increased the pErk/Erk ratio (70.05; *p* = 0.016; Tukey’s HSD) (Fig. 9b). Exposure to IFNγ (30 ng/ml) had no significant effect on the pErk/Erk ratio in hippocampal cell cultures, and IFNγ did not enhance the effect of IgG-IC on this outcome after 30 min nor after 24 h of exposure (Fig. 9b and Additional file 5).

**Fig. 10:**
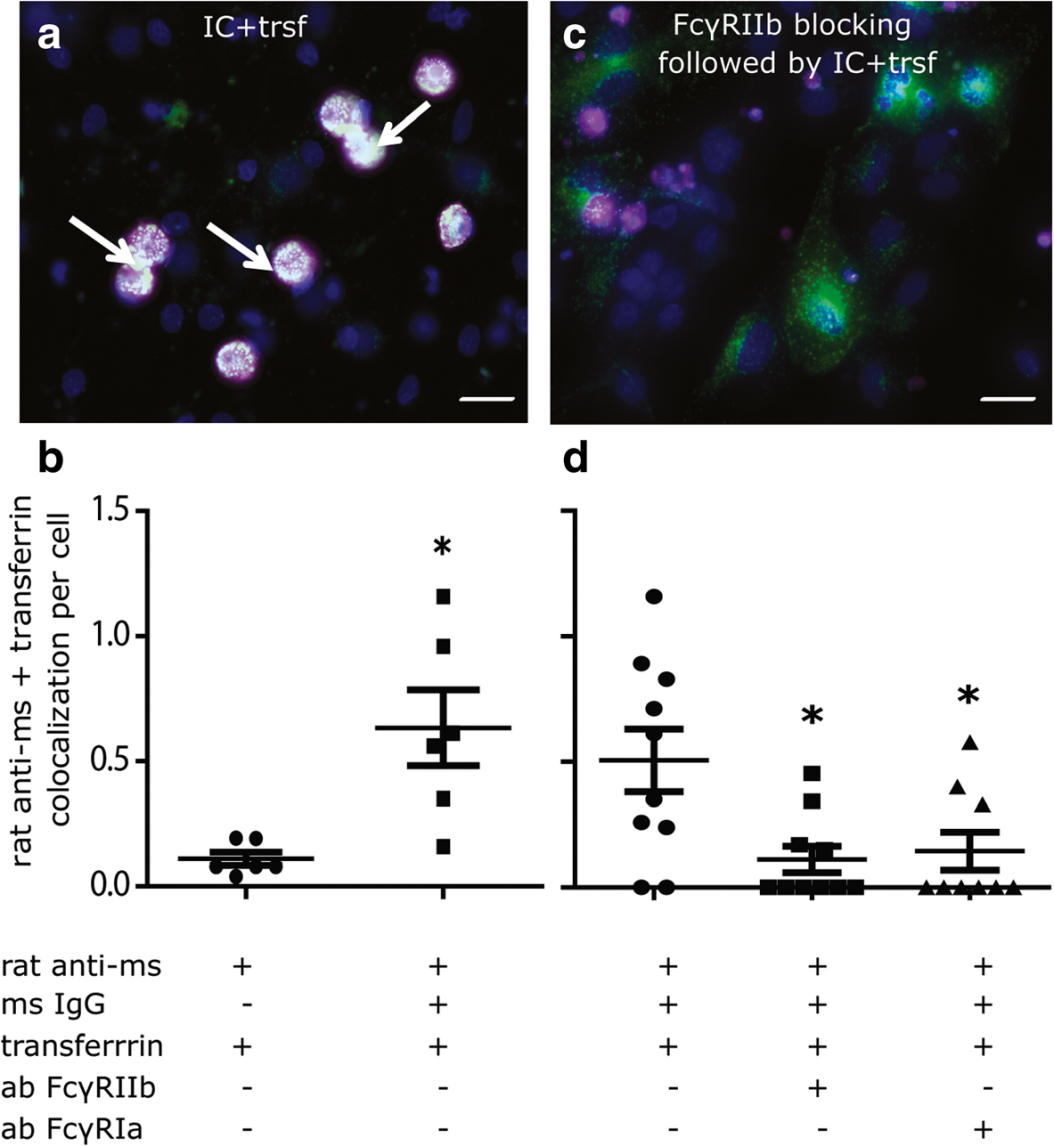
Exposure to IgG-immune complex (IC) triggers endocytosis of FcγR in rat hippocampal cells. **a**, **b** DIV 7 hippocampal cells were incubated with rat anti-mouse IgG (magenta), followed by incubation with normal mouse IgG and transferrin (green) to label the entire endosomal pathway; nuclei are stained blue (DAPI). **c**, **d** Cells were pre-incubated with either FcγRIIb or FcγRIa antibodies to block FcγR before incubation with rat anti-mouse IgG, mouse IgG, and transferrin. **a**, **c** Representative photomicrographs of co-localization of rat anti-mouse IgG and transferrin (evident as white puncta). **b**, **d** Quantification of dual-labeled puncta by *t* test of log-transformed data (**b**) or one-way ANOVA followed by Tukey’s post hoc test (**d**). Data are presented as the mean ± SEM. Scale bar = 30 μm. Trsf = transferrin; IC = IgG immune complex

In macrophages, cross-linking of FcγR by immune complex often results in FcγR internalization into endosomes [73]. To determine whether this also occurs in neural cells, DIV 7 hippocampal cell cultures were “primed” using rat anti-mouse IgG conjugated to AlexaFluor 647, followed by addition of mouse IgG and AlexaFluor 488-labeled rat transferrin, which labels the entire endosome pathway [55]. Intracellular puncta dual-labeled for rat anti-mouse IgG and transferrin (Fig. 10a, c) were quantified as a measure of FcγR internalization. The number of puncta immunopositive for both rat anti-mouse IgG and transferrin was significantly increased in cultures exposed to the IgG-IC (*p* = 0.011; df = 5; mean difference = −0.7416 by *t* test of log transformed data), but not in cultures exposed to rat anti-mouse IgG in the absence of mouse IgG (Fig. 10b). This effect of IgG-IC was significantly reduced in hippocampal cell cultures pre-incubated with antibodies against FcγRIa (CD64) or FcγRIIb (CD32) (*F* = 5.92; *p* = 0.007; df = 28; Tukey’s HSD) (Fig. 10c, d).

## Discussion

Emerging evidence suggests that FcγR are expressed by neurons and macroglia in the adult human and murine nervous system [7, 43]. However, available data are fragmented (see Additional file 6 which summarizes relevant information available in published scientific literature), and a comprehensive evaluation of FcγR expression and function in the developing brain is lacking. This also holds true for rat models of neurodevelopmental disease, despite the recognized value of rats as a model species in neurosciences and the increasing availability of novel genetic rat models of neurodevelopmental disease [74]. The data reported here address this gap by documenting in vivo expression of *FcgRIa*, *FcgRIIa*, *FcgRIIb*, *FcgRIIIa*, and *Fcgrt* mRNA in the neonatal rat cortex, hippocampus, and cerebellum at levels comparable to those observed in the liver and spleen. In vitro, expression of FcγRIa, FcγRIIb, and FcγRIIIa was confirmed at the mRNA and the protein level. In contrast, FcRn, which is encoded by *Fcgrt*, was not detected in either hippocampal or cortical cultures using either antibody-based or proteomic methods. A lack of correlation between mRNA and protein levels in biological samples is not unusual [75, 76], and to date, there is no published evidence of FcRn protein expression in neurons or astrocytes. However, it is possible that the lack of immunoreactivity for FcRn in our cultures could be due to technical limitations of the antibodies/mass spectrometry analyses used. Collectively, our data provide direct evidence that FcγRIa, FcγRIIb, and FcγRIIIa are expressed by developing hippocampal and cortical neurons and astrocytes, thereby proving that FcγR expression in the CNS is not limited to resident immune cells.

Consistent with what is known about FcγR signaling in immune cells [32, 77–79], we observed that IgG-IC triggered increased [Ca^2+^]_i_, Erk phosphorylation, and internalization of immune complexes in primary rat neuron-glia co-cultures. A potential confounding factor in the interpretation of these experiments is that the rat IgG used in the IgG-IC reacted with antigens other than FcγR on the surface of neurons and astrocytes. However, this possibility is not likely a significant issue because the rat IgG we used in our studies was highly cross-adsorbed against rat antigens. Moreover, antibodies against FcγRIa or FcγRIIb blocked immune complex internalization in neurons, consistent with evidence that both excitatory and inhibitory FcγR can internalize immune complexes in immune cells [33, 80–82].

A canonical response to cross-linking of FcγR by IgG-IC is increased [Ca^2+^]_i_. In primary hippocampal neurons exposed to IgG-IC, two general calcium responses were observed: a variable onset spike or a gradual rise in [Ca^2+^]_i_. These variable calcium responses to FcγR cross-linking are in contrast to the sharp calcium spikes reported in dorsal root ganglion (DRG) neurons exposed to a similar IgG-IC preparation [54]. The DRG calcium response resembles the rapid [Ca^2+^]_i_ spike typically observed in macrophages stimulated with IgG-opsonized erythrocytes [78]. However, the variable calcium responses observed in hippocampal neurons are in agreement with the variability of calcium responses in immune cells, which range from isolated [Ca^2+^]_i_ transients to long-lasting oscillations and increased plateaus [83, 84]. These variable calcium responses in immune cells are thought to be due to the diversity of FcγR subtypes expressed on these cells [34, 35, 85, 86]. Thus, the considerable variability observed in the calcium response of hippocampal cell cultures to IgG-IC likely reflects differences in the relative expression of activating versus inhibitory FcγR isotypes within and between cell types in our culture system, an interpretation that was confirmed by our flow cytometry data (co-expression of FcγRIa and FcγRIIb on the surface of neurons and astrocytes). Also consistent with this interpretation is that DRG neurons, unlike hippocampal neurons, do not express FcγRIIb or FcγRIIIa [54]. Interestingly, in primary hippocampal cell cultures, the individual components of the IgG-IC (mouse IgG or rat anti-mouse IgG) also elicited increased [Ca^2+^]_i_ when added at very high concentrations that are not physiologically relevant, suggesting that responsive cells in our cultures predominantly expressed the high affinity FcγRIa. This is supported also by the increased pErk/Erk ratio observed in response to the high concentration (100 μg/ml) of rat anti-mouse IgG. On the other hand, the lack of effect of exposure to the high concentration of IgG-IC on the pErk/Erk ratio, which was in contrast to the variable increase in [Ca]_i_ and the increased co-localization with transferrin at this concentration, is supported by evidence that [Ca]_i_ mobilization and FcγR internalization depend on the type and concentration of the stimulus, and both can occur independent of Erk phosphorylation [87–89]. Moreover, Erk activation in neurons is also dependent on the concentration of the stimulus [90]. These findings are further supported by evidence that calcium transients in immune cells can be differentially regulated by different FcγR [91] and that different FcγR have different affinities for different IgG isotypes. More sophisticated experiments with neurons knocked-down for individual FcγR subtypes and exposed to a low-to-high range of IgG-IC containing receptor-specific IgG isotypes could further elucidate the complex mechanisms likely underlying FcγR signaling in neurons and astrocytes in the developing brain.

FcγR expression in immune cells is regulated by IFNγ. Specifically, IFNγ upregulates FcγRIa expression [62, 92–94], stimulates FcγRIa function [92], and decreases the expression of FcγRIIb and FcRn [95, 96]. Similarly, we observed that IFNγ increased *FcgrIa* and decreased *FcgrIIb* and *Fcgrt* in DIV 2 and DIV 3 cortical cell cultures. We did not, however, see an effect of IFNγ on Erk phosphorylation after stimulation with IFNγ for 30 min. It has been reported that IFNγ upregulates macrophage FcγRIa at the protein level after prolonged (> 1 h) but not after short-term (30 min) stimulation [92]. However, even stimulation with IFNγ for 24 h did not significantly increase Erk phosphorylation in either hippocampal or cortical cell cultures (Additional file 5). While we cannot rule out the possibility that we did not sample at the appropriate time after IFNγ stimulation to detect increased Erk phosphorylation, our data do, nonetheless, support the hypothesis that IFNγ signaling plays a role in regulating *Fcgr* transcript expression in neurons and/or astrocytes in the developing rat brain.

The functional significance of FcγR expression in hippocampal and cortical neurons and astrocytes remains to be determined. Studies suggesting that FcγR trigger differentiation of oligodendrocyte precursor cells and are critical for normal development and function of Purkinje cells support a role for FcγR expressed on non-immune cells in CNS development [12, 13]. In support of this hypothesis, FcγRIa expression has been previously reported in human infant neocortical pyramidal neurons [97]. Conversely, FcγR may be involved in adverse neurodevelopmental outcomes [20–26], as suggested by evidence that inflammation can have detrimental effects in fetal brain development [98–102]. Recent evidence of a functional role for FcγR-bearing cells in antibody-dependent enhancement of Zika virus infection suggests a potentially critical role of FcγR expression on developing neurons and astrocytes in neurodevelopmental deficits resulting from infection [103, 104]. Alternatively, FcγR-mediated endocytosis of antibodies attached to viruses can also be part of a protective mechanism, as it is has been shown to be the first step in a TRIM21-mediated intracellular immune response which disables the virus in the cytosol [15, 105].

Considering the complexity of FcγR signaling that arises from the multiplicity of receptor subtypes/functions, their differing affinity for specific IgG subclasses, and their expression by multiple cell types, our novel data demonstrating developmental expression of functional FcγR by neurons and astrocytes and the regulation of their expression by classic immune molecules underscore the need for further characterization of the role of FcγR during normal and pathogenic neurodevelopment.

## Conclusions

The data reported herein constitute the first comprehensive investigation of FcγR expression and signaling on neurons and astrocytes of the developing rat brain. Our findings show that neurons and astrocytes in the developing brain express FcγR and that cross-linking of FcγR by IgG immune complexes activates downstream events in these non-immune cells, similar to events following FcγR activation in immune cells, including intracellular signaling and internalization of the antibody-receptor complex. These observations suggest a novel mechanism to explain the reported association between maternal antibodies that target antigens in the developing brain with increased risk for having a child diagnosed with a neurodevelopmental disorder.

## Additional files

Additional file 1: Developmental changes in *Fcgr* expression in the brain. Relative changes in Fcgr expression in the hippocampus, cortex, and cerebellum of postnatal day (PND) 7 Sprague Dawley rats compared to their PND1 littermates. (XLSX 10 kb)

Additional file 2: Developmental changes in *Fcgr* expression in the liver and spleen. Relative changes in *Fcgr* expression in the liver and spleen of postnatal day (PND) 7 Sprague Dawley rats compared to their PND1 littermates. (XLSX 10 kb)

Additional file 3: CD11b immunoreactivity in hippocampal and cortical cultures at DIV 0 and DIV 7. Hippocampal and cortical cells on DIV0 and DIV7 were immunolabeled for MAP2b (magenta), GFAP (green), and CD11b (orange). Almost no staining was observed for CD11b across both culture types and DIV. Quantification of CD11b staining on DIV 0 in hippocampal cells showed that fewer than 0.6% of cells were positive for CD11b (representative images of CD11b positive cells are shown here). (JPEG 1340 kb)

Additional file 4: Percentage of MAP2b and GFAP, and CD11b cells in hippocampi cell cultures at DIV 0. Dissociated hippocampal cell cultures were plated and fixed at DIV 0 to immunostain for MAP2b, GFAP, and CD11b. Immunoreactivity was imaged using the ImageXpress high-content imaging system; and the number of immunoreactive cells was quantified using the Custom Module Editor in the MetaXpress Software (Molecular Devices). (JPEG 219 kb)

Additional file 5: IFNγ does not affect Erk phosphorylation in primary neuronal cell cultures. DIV 7 hippocampal and cortical cell cultures were exposed for 24 h to varying concentrations of IgG-IC (10 or 100 μg/ml) or rat anti-mouse IgG (10 or 100 μg/ml) in the presence or absence of 30 ng/ml IFNγ. Cell lysates were separated by SDS PAGE and immunoblotted for pErk, total Erk, and GAPDH. The optical density of bands immunoreactive for pErk and total Erk was normalized to the optical density of GAPDH immunoreactive bands from the same sample. The ratio of pErk to Erk is plotted as a percentage of vehicle controls. Data from a single replicate per condition in one experiment. r@m: rat anti-mouse IgG; IC: IgG-IC immune complex. (PDF 403 kb)

Additional file 6: Summary of the published literature documenting FcγR expression in neurons and macroglia**.** Tabulated summary of evidence from the published literature for expression of FcγR in neurons and macroglia in the central and peripheral nervous system in rodents and humans. (XLSX 13 kb)

## Abbreviations

araC: Cytosine-D-arabinofuranoside
AUC: Area under the curve
BSA: Bovine serum albumin
CD: Cluster of differentiation
CSC: Cell surface capture
DIV: Day in vitro
DRG: Dorsal root ganglion
FBS: Fetal bovine serum
Fc: Fragment crystallizable
FcRn: Neonatal Fc receptor
FcγR: Fc gamma receptor
IC: Immune complex
GAPDH: Glyceraldehyde 3-phosphate dehydrogenase
HCD: High-energy-induced collisional dissociation
HPLC: High-performance liquid chromatography
IFNγ: Interferon gamma
IgG: Immunoglobulin
ITAM: Immunoreceptor tyrosine-based activation motif
ITIM: Immunoreceptor tyrosine-based inhibition motif
MS: Mass spectrometry
NK cell: Natural killer cell
PBS: Phosphate buffered saline
PE: Phycoerythrin
pErk: Phosphorylated extracellular signal-regulated kinase
PFA: Paraformaldehyde
qPCR: Quantitative polymerase chain reaction
ROI: Region of interest
SDS: Sodium dodecyl sulfate
TPP: Trans-proteomic pipeline

## References

[1] Stephan AH, Barres BA, Stevens B (2012). The complement system: an unexpected role in synaptic pruning during development and disease. Annu Rev Neurosci.

[2] McAllister AK (2014). Major histocompatibility complex I in brain development and schizophrenia. Biol Psychiatry.

[3] Marin I, Kipnis J (2013). Learning and memory ... and the immune system. Learn Mem.

[4] Lian H, Yang L, Cole A, Sun L, Chiang AC, Fowler SW, Shim DJ, Rodriguez-Rivera J, Taglialatela G, Jankowsky JL (2015). NFkappaB-activated astroglial release of complement C3 compromises neuronal morphology and function associated with Alzheimer’s disease. Neuron.

[5] Gorelik A, Sapir T, Haffner-Krausz R, Olender T, Woodruff TM, Reiner O. Developmental activities of the complement pathway in migrating neurons. 2017;8:15096. 10.1038/ncomms15096 PMC5418580 28462915

[6] Cebrián C, Loike JD, Sulzer D (2014). Neuronal MHC-I expression and its implications in synaptic function, axonal regeneration and Parkinson’s and other brain diseases. Front Neuroanat.

[7] Fuller JP, Stavenhagen JB, Teeling JL (2014). New roles for Fc receptors in neurodegeneration-the impact on immunotherapy for Alzheimer’s disease. Front Neurosci.

[8] Jiang H, Shen X, Chen Z, Liu F, Wang T, Xie Y, Ma C (2017). Nociceptive neuronal Fc-gamma receptor I is involved in IgG immune complex induced pain in the rat. Brain Behav Immun.

[9] Qu L (2012). Neuronal Fc gamma receptor I as a novel mediator for IgG immune complex-induced peripheral sensitization. Neural Regen Res.

[10] Suemitsu S, Watanabe M, Yokobayashi E, Usui S, Ishikawa T, Matsumoto Y, Yamada N, Okamoto M, Kuroda S (2010). Fcgamma receptors contribute to pyramidal cell death in the mouse hippocampus following local kainic acid injection. Neuroscience.

[11] Kam T-I, Song S, Gwon Y, Park H, Yan J-J, Im I, Choi J-W, Choi T-Y, Kim J, Song D-K (2013). FcγRIIb mediates amyloid-β neurotoxicity and memory impairment in Alzheimer’s disease. J Clin Invest.

[12] Nakahara J, Tan-Takeuchi K, Seiwa C, Gotoh M, Kaifu T, Ujike A, Inui M, Yagi T, Ogawa M, Aiso S (2003). Signaling via immunoglobulin Fc receptors induces oligodendrocyte precursor cell differentiation. Dev Cell.

[13] Nakamura K, Hirai H, Torashima T, Miyazaki T, Tsurui H, Xiu Y, Ohtsuji M, Lin QS, Tsukamoto K, Nishimura H (2007). CD3 and immunoglobulin G Fc receptor regulate cerebellar functions. Mol Cell Biol.

[14] Congdon EE, Gu J, Sait HBR, Sigurdsson EM (2013). Antibody uptake into neurons occurs primarily via Clathrin-dependent Fcγ receptor endocytosis and is a prerequisite for acute tau protein clearance. J Biol Chem.

[15] Kondo A, Shahpasand K, Mannix R, Qiu J, Moncaster J, Chen C-H, Yao Y, Lin Y-M, Driver JA, Sun Y (2015). Antibody against early driver of neurodegeneration cis P-tau blocks brain injury and tauopathy. Nature.

[16] Strous RD, Shoenfeld Y (2006). Schizophrenia, autoimmunity and immune system dysregulation: a comprehensive model updated and revisited. J Autoimmun.

[17] Brown AS (2011). The environment and susceptibility to schizophrenia. Prog Neurobiol.

[18] Knuesel I, Chicha L, Britschgi M, Schobel SA, Bodmer M, Hellings JA, Toovey S, Prinssen EP (2014). Maternal immune activation and abnormal brain development across CNS disorders. Nat Rev Neurol.

[19] Fox-Edmiston E, Van de Water J (2015). Maternal anti-fetal brain IgG autoantibodies and autism spectrum disorder: current knowledge and its implications for potential therapeutics. CNS Drugs.

[20] Dalton P, Deacon R, Blamire A, Pike M, McKinlay I, Stein J, Styles P, Vincent A (2003). Maternal neuronal antibodies associated with autism and a language disorder. Ann Neurol.

[21] Zimmerman AW, Connors SL, Matteson KJ, Lee LC, Singer HS, Castaneda JA, Pearce DA (2007). Maternal antibrain antibodies in autism. Brain Behav Immun.

[22] Braunschweig D, Ashwood P, Krakowiak P, Hertz-Picciotto I, Hansen R, Croen LA, Pessah IN, Van de Water J (2008). Autism: maternally derived antibodies specific for fetal brain proteins. Neurotoxicology.

[23] Martinez-Cerdeno V, Camacho J, Fox E, Miller E, Ariza J, Kienzle D, Plank K, Noctor SC, Van de Water J (2016). Prenatal exposure to autism-specific maternal autoantibodies alters proliferation of cortical neural precursor cells, enlarges brain, and increases neuronal size in adult animals. Cereb Cortex.

[24] Nordahl CW, Braunschweig D, Iosif AM, Lee A, Rogers S, Ashwood P, Amaral DG, Van de Water J (2013). Maternal autoantibodies are associated with abnormal brain enlargement in a subgroup of children with autism spectrum disorder. Brain Behav Immun.

[25] Braunschweig D, Krakowiak P, Duncanson P, Boyce R, Hansen RL, Ashwood P, Hertz-Picciotto I, Pessah IN, Van de Water J (2013). Autism-specific maternal autoantibodies recognize critical proteins in developing brain. Transl Psychiatry.

[26] Bauman MD, Iosif AM, Ashwood P, Braunschweig D, Lee A, Schumann CM, Van de Water J, Amaral DG (2013). Maternal antibodies from mothers of children with autism alter brain growth and social behavior development in the rhesus monkey. Transl Psychiatry.

[27] Brzustowicz LM, Hayter JE, Hodgkinson KA, Chow EW, Bassett AS (2002). Fine mapping of the schizophrenia susceptibility locus on chromosome 1q22. Hum Hered.

[28] Theoharides TC, Zhang B (2011). Neuro-inflammation, blood-brain barrier, seizures and autism. J Neuroinflammation.

[29] de Smith AJ, Tsalenko A, Sampas N, Scheffer A, Yamada NA, Tsang P, Ben-Dor A, Yakhini Z, Ellis RJ, Bruhn L (2007). Array CGH analysis of copy number variation identifies 1284 new genes variant in healthy white males: implications for association studies of complex diseases. Hum Mol Genet.

[30] Hulett MD, Hogarth PM (1994). Molecular basis of Fc receptor function. Adv Immunol.

[31] Berken A, Benacerraf B (1966). Properties of antibodies cytophilic for macrophages. J Exp Med.

[32] Nimmerjahn F, Ravetch JV (2008). Fcgamma receptors as regulators of immune responses. Nat Rev Immunol.

[33] Guilliams M, Bruhns P, Saeys Y, Hammad H, Lambrecht BN (2014). The function of Fcgamma receptors in dendritic cells and macrophages. Nat Rev Immunol.

[34] Rosales C, Uribe-Querol E (2013). Fc receptors: cell activators of antibody functions. Adv Biosci Biotechnol.

[35] Hogarth PM, Pietersz GA (2012). Fc receptor-targeted therapies for the treatment of inflammation, cancer and beyond. Nat Rev Drug Discov.

[36] Simister NE, Mostov KE (1989). An Fc receptor structurally related to MHC class I antigens. Nature.

[37] Linnartz B, Neumann H (2013). Microglial activatory (immunoreceptor tyrosine-based activation motif)- and inhibitory (immunoreceptor tyrosine-based inhibition motif)-signaling receptors for recognition of the neuronal glycocalyx. Glia.

[38] Brown GC, Neher JJ (2014). Microglial phagocytosis of live neurons. Nat Rev Neurosci.

[39] Cahoy JD, Emery B, Kaushal A, Foo LC, Zamanian JL, Christopherson KS, Xing Y, Lubischer JL, Krieg PA, Krupenko SA (2008). A transcriptome database for astrocytes, neurons, and oligodendrocytes: a new resource for understanding brain development and function. J Neurosci.

[40] Sharma K, Schmitt S, Bergner CG, Tyanova S, Kannaiyan N, Manrique-Hoyos N, Kongi K, Cantuti L, Hanisch UK, Philips MA (2015). Cell type- and brain region-resolved mouse brain proteome. Nat Neurosci.

[41] Li YN, Qin XJ, Kuang F, Wu R, Duan XL, Ju G, Wang BR (2008). Alterations of Fc gamma receptor I and toll-like receptor 4 mediate the antiinflammatory actions of microglia and astrocytes after adrenaline-induced blood-brain barrier opening in rats. J Neurosci Res.

[42] Kam TI, Song S, Gwon Y, Park H, Yan JJ, Im I, Choi JW, Choi TY, Kim J, Song DK (2013). FcgammaRIIb mediates amyloid-beta neurotoxicity and memory impairment in Alzheimer’s disease. J Clin Invest.

[43] Okun E, Mattson MP, Arumugam TV (2010). Involvement of Fc receptors in disorders of the central nervous system. NeuroMolecular Med.

[44] Fernandez-Vizarra P, Lopez-Franco O, Mallavia B, Higuera-Matas A, Lopez-Parra V, Ortiz-Munoz G, Ambrosio E, Egido J, Gomez-Guerrero C, Almeida OF (2012). Immunoglobulin G Fc receptor deficiency prevents Alzheimer-like pathology and cognitive impairment in mice. Brain.

[45] Wayman GA, Impey S, Marks D, Saneyoshi T, Grant WF, Derkach V, Soderling TR (2006). Activity-dependent dendritic arborization mediated by CaM-kinase I activation and enhanced CREB-dependent transcription of Wnt-2. Neuron.

[46] Grodzki AC, Poola B, Pasupuleti N, Nantz MH, Lein PJ, Gorin F, Novel Carboline A (2015). Derivative inhibits nitric oxide formation in macrophages independent of effects on tumor necrosis factor alpha and interleukin-1beta expression. J Pharmacol Exp Ther.

[47] Donnelly S, O'Neill SM, Sekiya M, Mulcahy G, Dalton JP (2005). Thioredoxin peroxidase secreted by Fasciola hepatica induces the alternative activation of macrophages. Infect Immun.

[48] Stamou M, Wu X, Kania-Korwel I, Lehmler HJ, Lein PJ (2014). Cytochrome p450 mRNA expression in the rodent brain: species-, sex-, and region-dependent differences. Drug Metab Dispos.

[49] Pfaffl MW, Horgan GW, Dempfle L (2002). Relative expression software tool (REST) for group-wise comparison and statistical analysis of relative expression results in real-time PCR. Nucleic Acids Res.

[50] Santos AR, Duarte CB (2008). Validation of internal control genes for expression studies: effects of the neurotrophin BDNF on hippocampal neurons. J Neurosci Res.

[51] Pfaffl MW (2001). A new mathematical model for relative quantification in real-time RT-PCR. Nucleic Acids Res.

[52] Nelissen K, Smeets K, Fau-Mulder M, Mulder M, Fau-Hendriks JJA, Hendriks JJ, Fau-Ameloot M, Ameloot M. Selection of reference genes for gene expression studies in rat oligodendrocytes using quantitative real time PCR. J Neurosci Methods. 2010;187:78-83. 10.1016/j.jneumeth.2009.12.018 20036692

[53] Wollscheid B, Bausch-Fluck D, Henderson C, O'Brien R, Bibel M, Schiess R, Aebersold R, Watts JD (2009). Mass-spectrometric identification and relative quantification of N-linked cell surface glycoproteins. Nat Biotechnol.

[54] Qu L, Zhang P, LaMotte RH, Ma C (2011). Neuronal Fc-gamma receptor I mediated excitatory effects of IgG immune complex on rat dorsal root ganglion neurons. Brain Behav Immun.

[55] Goebl NA, Babbey CM, Datta-Mannan A, Witcher DR, Wroblewski VJ, Dunn KW (2008). Neonatal Fc receptor mediates internalization of Fc in transfected human endothelial cells. Mol Biol Cell.

[56] Bouchard JF, Moore SW, Tritsch NX, Roux PP, Shekarabi M, Barker PA, Kennedy TE (2004). Protein kinase a activation promotes plasma membrane insertion of DCC from an intracellular pool: a novel mechanism regulating commissural axon extension. J Neurosci.

[57] Carrodus NL, Teng KS, Munro KM, Kennedy MJ, Gunnersen JM. Differential labeling of cell-surface and internalized proteins after antibody feeding of live cultured neurons. J Vis Exp. 2014;(84):e51139. 10.3791/51139 PMC4123487 24561550

[58] Simms HH, D'Amico R (1994). Regulation of intracellular polymorphonuclear leukocyte Fc receptors by lipopolysaccharide. Cell Immunol.

[59] Omasits U, Ahrens CH, Müller S, Wollscheid B (2014). Protter: interactive protein feature visualization and integration with experimental proteomic data. Bioinformatics.

[60] Fensterl V, Sen GC (2009). Interferons and viral infections. Biofactors.

[61] Aittomaki S, Yang J, Scott EW, Simon MC, Silvennoinen O (2002). Distinct functions for signal transducer and activator of transcription 1 and PU.1 in transcriptional activation of Fc gamma receptor I promoter. Blood.

[62] Okayama Y, Kirshenbaum AS, Metcalfe DD (2000). Expression of a functional high-affinity IgG receptor, Fc gamma RI, on human mast cells: up-regulation by IFN-gamma. J Immunol.

[63] Guyre PM, Morganelli PM, Miller R (1983). Recombinant immune interferon increases immunoglobulin G Fc receptors on cultured human mononuclear phagocytes. J Clin Invest.

[64] Erbe DV, Collins JE, Shen L, Graziano RF, Fanger MW (1990). The effect of cytokines on the expression and function of Fc receptors for IgG on human myeloid cells. Mol Immunol.

[65] Woodroofe MN, Hayes GM, Cuzner ML (1989). Fc receptor density, MHC antigen expression and superoxide production are increased in interferon-gamma-treated microglia isolated from adult rat brain. Immunology.

[66] Mizuno T, Zhang G, Takeuchi H, Kawanokuchi J, Wang J, Sonobe Y, Jin S, Takada N, Komatsu Y, Suzumura A (2008). Interferon-gamma directly induces neurotoxicity through a neuron specific, calcium-permeable complex of IFN-gamma receptor and AMPA GluR1 receptor. FASEB J.

[67] Mohamed HA, Mosier DR, Zou LL, Siklos L, Alexianu ME, Engelhardt JI, Beers DR, Le WD, Appel SH (2002). Immunoglobulin Fc gamma receptor promotes immunoglobulin uptake, immunoglobulin-mediated calcium increase, and neurotransmitter release in motor neurons. J Neurosci Res.

[68] Andoh T, Kuraishi Y (2004). Direct action of immunoglobulin G on primary sensory neurons through Fc gamma receptor I. FASEB J.

[69] Cao Z, Hammock BD, McCoy M, Rogawski MA, Lein PJ, Pessah IN (2012). Tetramethylenedisulfotetramine alters Ca(2)(+) dynamics in cultured hippocampal neurons: mitigation by NMDA receptor blockade and GABA(A) receptor-positive modulation. Toxicol Sci.

[70] Tanaka T, Saito H, Matsuki N (1996). Intracellular calcium oscillation in cultured rat hippocampal neurons: a model for glutamatergic neurotransmission. Jpn J Pharmacol.

[71] van Mirre E, Teeling JL, van der Meer JWM, Bleeker WK, Hack CE (2004). Monomeric IgG in intravenous Ig preparations is a functional antagonist of FcγRII and FcγRIIIb. J Immunol.

[72] Tanaka M, Krutzik SR, Sieling PA, Lee D, Rea TH, Modlin RL (2009). Activation of FcγR1 on monocytes triggers differentiation into immature dendritic cells that induce autoreactive T cell responses. J Immunol (Baltimore, Md: 1950).

[73] Mellman I, Plutner H (1984). Internalization and degradation of macrophage Fc receptors bound to polyvalent immune complexes. J Cell Biol.

[74] Ellenbroek B, Youn J (2016). Rodent models in neuroscience research: is it a rat race?. Dis Model Mech.

[75] Maier T, Guell M, Serrano L (2009). Correlation of mRNA and protein in complex biological samples. FEBS Lett.

[76] Liu Y, Beyer A, Aebersold R (2016). On the dependency of cellular protein levels on mRNA abundance. Cell.

[77] Young JD, Ko SS, Cohn ZA (1984). The increase in intracellular free calcium associated with IgG gamma 2b/gamma 1 Fc receptor-ligand interactions: role in phagocytosis. Proc Natl Acad Sci U S A.

[78] Myers JT, Swanson JA (2002). Calcium spikes in activated macrophages during Fcgamma receptor-mediated phagocytosis. J Leukoc Biol.

[79] Lucas M, Zhang X, Prasanna V, Mosser DM (2005). ERK activation following macrophage FcgammaR ligation leads to chromatin modifications at the IL-10 locus. J Immunol.

[80] Amigorena S, Bonnerot C (1999). Fc receptor signaling and trafficking: a connection for antigen processing. Immunol Rev.

[81] Miettinen HM, Matter K, Hunziker W, Rose JK, Mellman I (1992). Fc receptor endocytosis is controlled by a cytoplasmic domain determinant that actively prevents coated pit localization. J Cell Biol.

[82] Zhang CY, Booth JW (2011). Differences in endocytosis mediated by FcgammaRIIA and FcgammaRIIB2. Mol Immunol.

[83] Oh-hora M (2009). Calcium signaling in the development and function of T-lineage cells. Immunol Rev.

[84] Vig M, Kinet JP (2009). Calcium signaling in immune cells. Nat Immunol.

[85] Attout T, Floto A, Launay P (2014). Calcium channels in Fc receptor signaling. Curr Top Microbiol Immunol.

[86] Nunes P, Demaurex N (2010). The role of calcium signaling in phagocytosis. J Leukoc Biol.

[87] Ren L, Campbell A, Fang H, Gautam S, Elavazhagan S, Fatehchand K, Mehta P, Stiff A, Reader BF, Mo X (2016). Analysis of the effects of the Bruton’s tyrosine kinase (Btk) inhibitor ibrutinib on monocyte Fcγ receptor (FcγR) function. J Biol Chem.

[88] Yang L, Mao L, Tang Q, Samdani S, Liu Z, Wang JQ (2004). A novel Ca&lt;sup&gt;2+&lt;/sup&gt;-independent signaling pathway to extracellular signal-regulated protein kinase by coactivation of NMDA receptors and metabotropic glutamate receptor 5 in neurons. J Neurosci.

[89] Ganesan LP, Joshi T, Fang H, Kutala VK, Roda J, Trotta R, Lehman A, Kuppusamy P, Byrd JC, Carson WE (2006). FcγR-induced production of superoxide and inflammatory cytokines is differentially regulated by SHIP through its influence on PI3K and/or Ras/Erk pathways. Blood.

[90] Dougherty MK, Ritt DA, Zhou M, Specht SI, Monson DM, Veenstra TD, Morrison DK (2009). KSR2 is a calcineurin substrate that promotes ERK cascade activation in response to calcium signals. Mol Cell.

[91] Edberg JC, Moon JJ, Chang DJ, Kimberly RP (1998). Differential regulation of human neutrophil FcγRIIa (CD32) and FcγRIIIb (CD16)-induced Ca2+ transients. J Biol Chem.

[92] Bezbradica JS, Rosenstein RK, DeMarco RA, Brodsky I, Medzhitov R (2014). A role for the ITAM signaling module in specifying cytokine-receptor functions. Nat Immunol.

[93] Pearse RN, Feinman R, Ravetch JV (1991). Characterization of the promoter of the human gene encoding the high-affinity IgG receptor: transcriptional induction by gamma-interferon is mediated through common DNA response elements. Proc Natl Acad Sci U S A.

[94] Platanias LC (2005). Mechanisms of type-I- and type-II-interferon-mediated signalling. Nat Rev Immunol.

[95] Liu X, Ye L, Bai Y, Mojidi H, Simister NE, Zhu X (2008). Activation of the JAK/STAT-1 signaling pathway by IFN-gamma can down-regulate functional expression of the MHC class I-related neonatal Fc receptor for IgG. J Immunol.

[96] Boruchov AM, Heller G, Veri M-C, Bonvini E, Ravetch JV, Young JW (2005). Activating and inhibitory IgG Fc receptors on human DCs mediate opposing functions. J Clin Invest.

[97] Bouras C, Riederer BM, Hof PR, Giannakopoulos P (2003). Induction of MC-1 immunoreactivity in axons after injection of the Fc fragment of human immunoglobulins in macaque monkeys. Acta Neuropathol.

[98] Hagberg H, Gressens P, Mallard C (2012). Inflammation during fetal and neonatal life: implications for neurologic and neuropsychiatric disease in children and adults. Ann Neurol.

[99] Angelidou A, Asadi S, Alysandratos KD, Karagkouni A, Kourembanas S, Theoharides TC (2012). Perinatal stress, brain inflammation and risk of autism-review and proposal. BMC Pediatr.

[100] Stolp HB, Dziegielewska KM (2009). Review: role of developmental inflammation and blood-brain barrier dysfunction in neurodevelopmental and neurodegenerative diseases. Neuropathol Appl Neurobiol.

[101] Meyer U, Feldon J, Dammann O (2011). Schizophrenia and autism: both shared and disorder-specific pathogenesis via perinatal inflammation?. Pediatr Res.

[102] Weir RK, Forghany R, Smith SE, Patterson PH, McAllister AK, Schumann CM, Bauman MD. Preliminary evidence of neuropathology in nonhuman primates prenatally exposed to maternal immune activation. Brain Behav Immun. 2015;48:139-46. 10.1016/j.bbi.2015.03.009 PMC5671487 25816799

[103] Priyamvada L, Quicke KM, Hudson WH, Onlamoon N, Sewatanon J, Edupuganti S, Pattanapanyasat K, Chokephaibulkit K, Mulligan MJ, Wilson PC (2016). Human antibody responses after dengue virus infection are highly cross-reactive to Zika virus. Proc Natl Acad Sci U S A.

[104] Miner Jonathan J, Diamond Michael S (2016). Understanding how Zika virus enters and infects neural target cells. Cell Stem Cell.

[105] Mallery DL, McEwan WA, Bidgood SR, Towers GJ, Johnson CM, James LC (2010). Antibodies mediate intracellular immunity through tripartite motif-containing 21 (TRIM21). Proc Natl Acad Sci U S A.

[106] Vizcaino JA, Csordas A, Del-Toro N, Dianes JA, Griss J, Lavidas I, Mayer G, Perez-Riverol Y, Reisinger F, Ternent T (2016). 2016 update of the PRIDE database and its related tools. Nucleic Acids Res.

