# Supplementary figures and images for: Fc gamma receptors are expressed in the developing rat brain and activate downstream signaling molecules upon cross-linking with immune complex
Source: J Neuroinflammation. 2018 Jan 6;15:7. doi: 10.1186/s12974-017-1050-z (PMC5756609; doi:10.1186/s12974-017-1050-z)

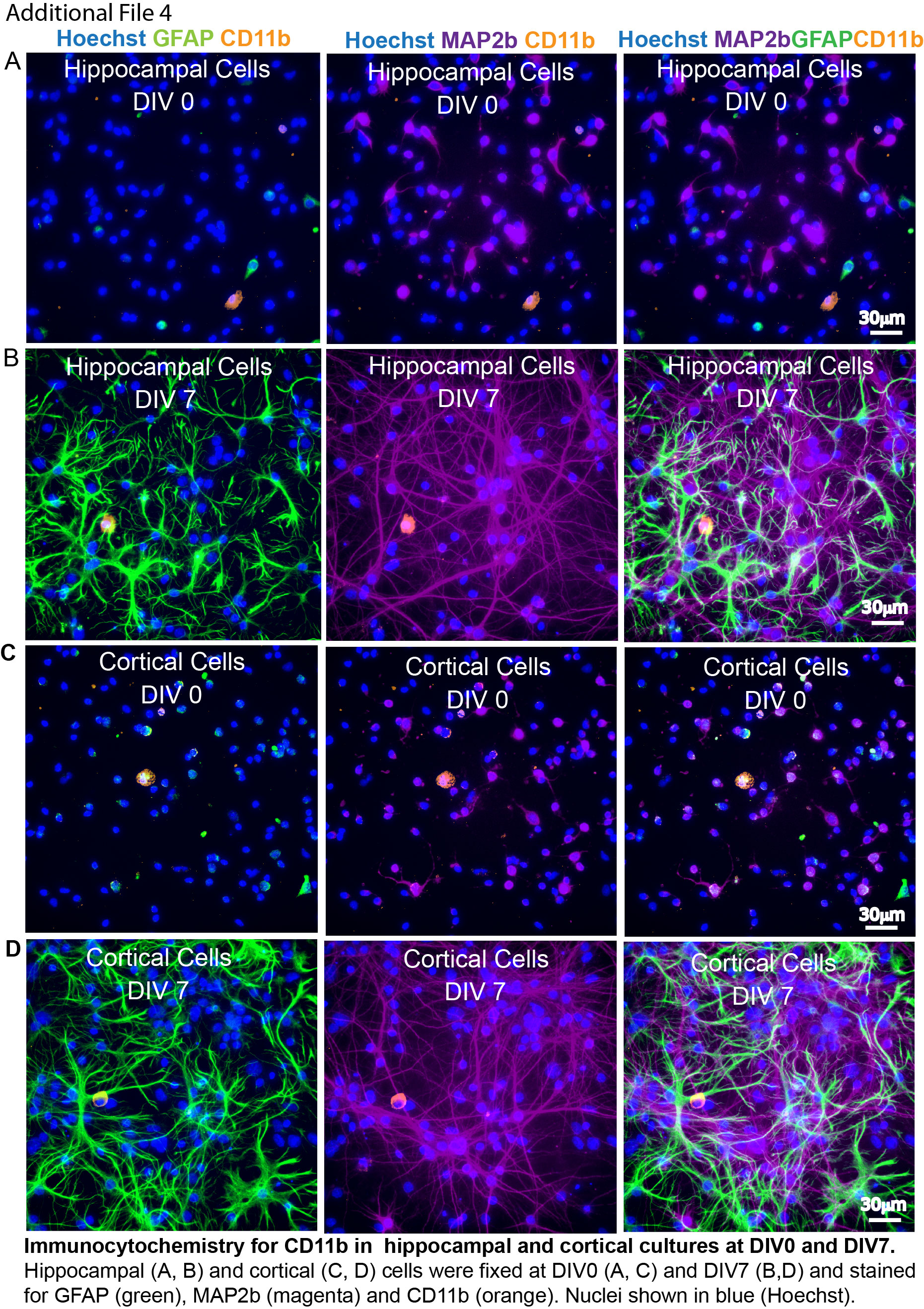

Supplement: Supplementary file 4 — Percentage of MAP2b and GFAP, and CD11b cells in hippocampi cell cultures at DIV 0. Dissociated hippocampal cell cultures were plated and fixed at DIV 0 to immunostain for MAP2b, GFAP, and CD11b. Immunoreactivity was imaged using the ImageXpress high-content imaging system; and the number of immunoreactive cells was quantified using the Custom Module Editor in the MetaXpress Software (Molecular Devices). (JPEG 219 kb) [file 12974_2017_1050_MOESM4_ESM.jpg]

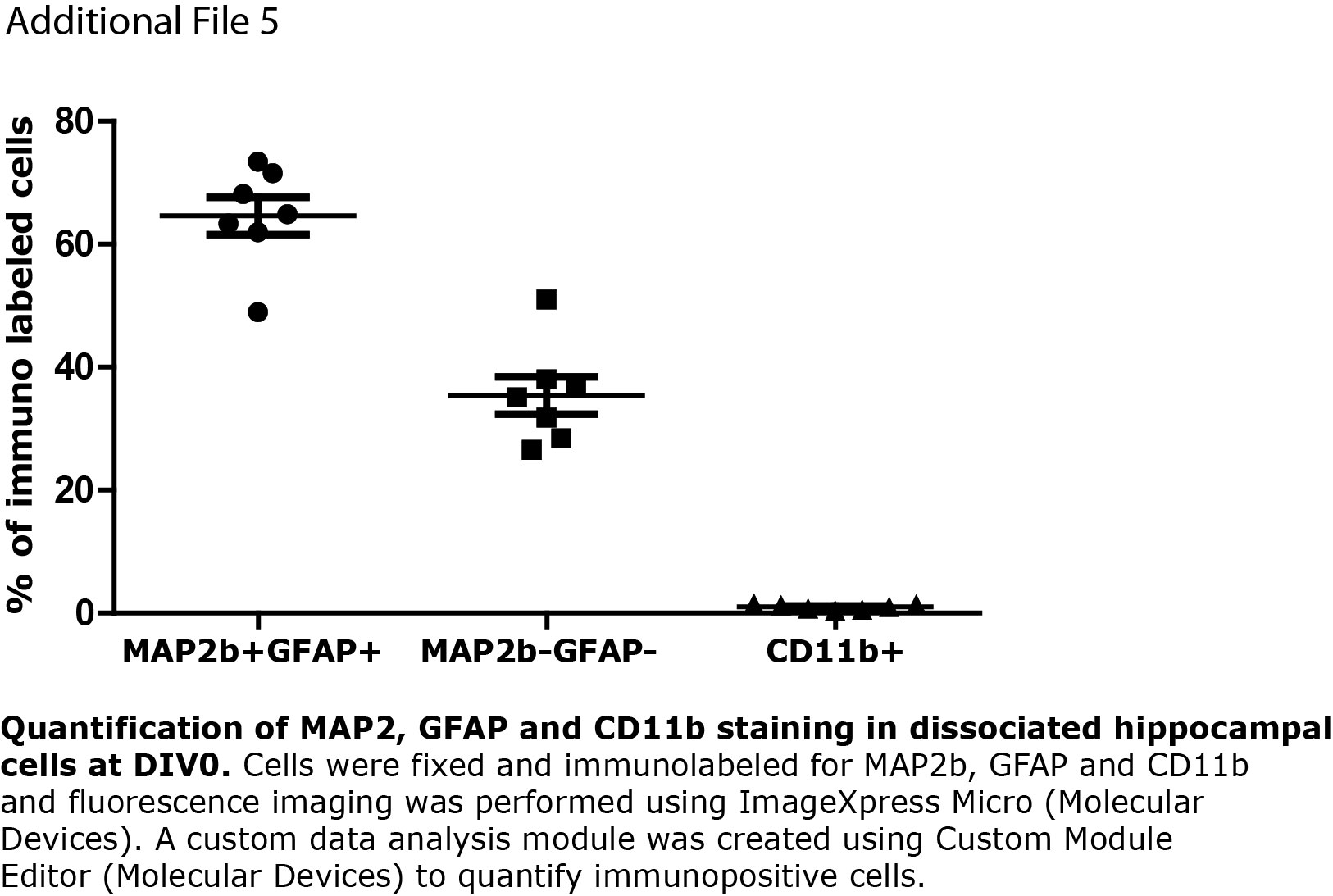

Supplement: Supplementary file 5 — IFNγ does not affect Erk phosphorylation in primary neuronal cell cultures. DIV 7 hippocampal and cortical cell cultures were exposed for 24 h to varying concentrations of IgG-IC (10 or 100 μg/ml) or rat anti-mouse IgG (10 or 100 μg/ml) in the presence or absence of 30 ng/ml IFNγ. Cell lysates were separated by SDS PAGE and immunoblotted for pErk, total Erk, and GAPDH. The optical density of bands immunoreactive for pErk and total Erk was normalized to the optical density of GAPDH immunoreactive bands from the same sample. The ratio of pErk to Erk is plotted as a percentage of vehicle controls. Data from a single replicate per condition in one experiment. r@m: rat anti-mouse IgG; IC: IgG-IC immune complex. (PDF 403 kb) [file 12974_2017_1050_MOESM5_ESM.jpg]
