# Supplementary material for: Fc gamma receptors are expressed in the developing rat brain and activate downstream signaling molecules upon cross-linking with immune complex
Source: J Neuroinflammation. 2018 Jan 6;15:7. doi: 10.1186/s12974-017-1050-z (PMC5756609; doi:10.1186/s12974-017-1050-z)
Supplement: Supplementary file 6 — Summary of the published literature documenting FcγR expression in neurons and macroglia. Tabulated summary of evidence from the published literature for expression of FcγR in neurons and macroglia in the central and peripheral nervous system in rodents and humans. (XLSX 13 kb) [file 12974_2017_1050_MOESM6_ESM.pdf]

Additional File 6

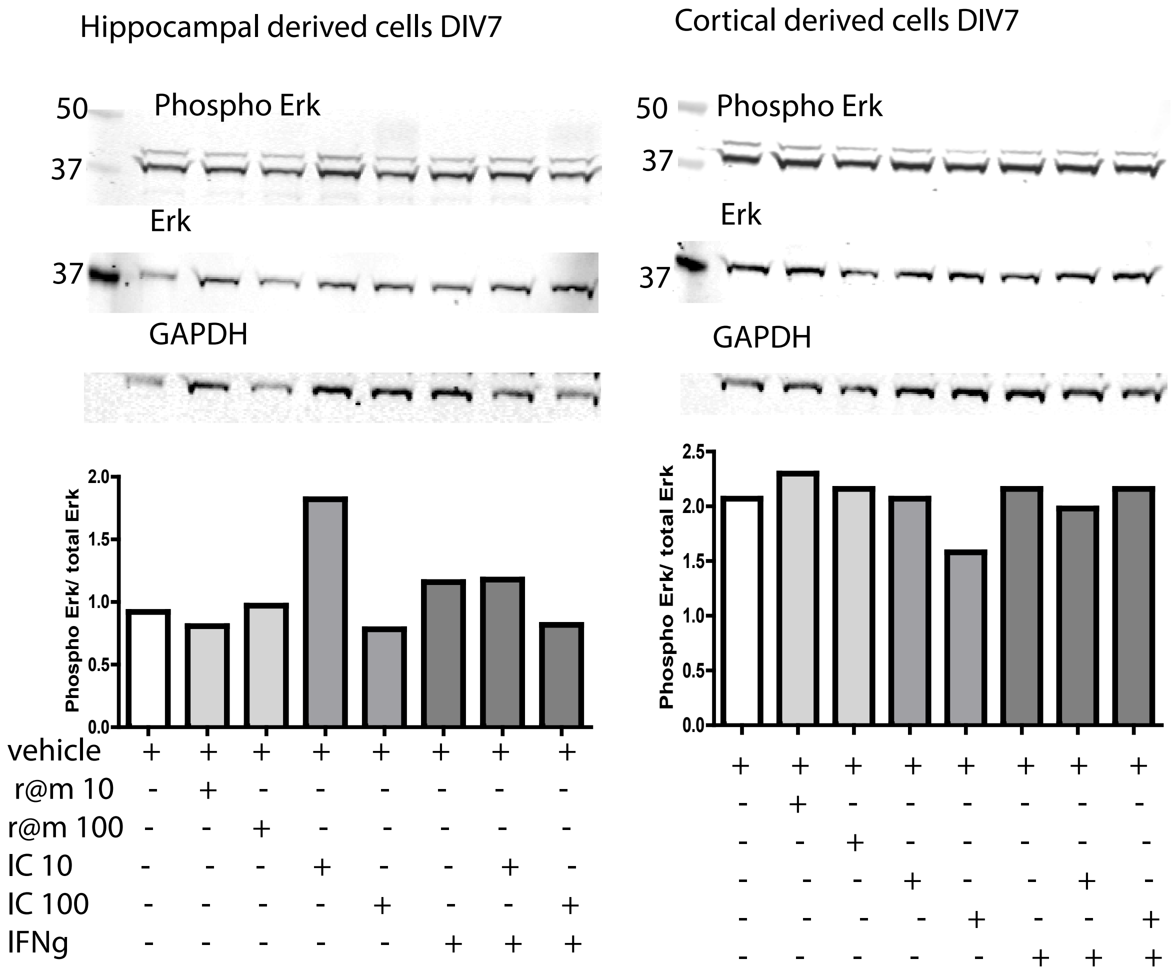

Erk Phosphorylation after 24h exposure to ImmunoComplexes (IC) and/or IFNg. DIV7 cortical and hippocampal cells stimulated for 24h with Immuno complexes at 10 and 100ng/m and IFNg 30ng/ml did not increase the MAPKinase Erk phosphorylation.
